# The Role of Endocrine Disruptors Bisphenols and Phthalates in Obesity: Current Evidence, Perspectives and Controversies

**DOI:** 10.3390/ijms25010675

**Published:** 2024-01-04

**Authors:** Maria Dalamaga, Dimitrios Kounatidis, Dimitrios Tsilingiris, Natalia G. Vallianou, Irene Karampela, Sotiria Psallida, Athanasios G. Papavassiliou

**Affiliations:** 1 Department of Biological Chemistry, Medical School, National and Kapodistrian University of Athens, 11527 Athens, Greece; 2 Department of Internal Medicine, ‘Evangelismos’ General Hospital, 10676 Athens, Greece; dimitriskounatidis82@outlook.com (D.K.); natalia.vallianou@hotmail.com (N.G.V.); 3 First Department of Internal Medicine, University Hospital of Alexandroupolis, Democritus University of Thrace, 68100 Alexandroupolis, Greece; tsilingirisd@gmail.com; 4 Second Department of Critical Care, ‘Attikon’ General University Hospital, Medical School, National and Kapodistrian University of Athens, 12462 Athens, Greece; eikaras1@gmail.com; 5 Department of Microbiology, ‘KAT’ General Hospital of Attica, 14561 Athens, Greece; psallidasotiria@gmail.com

**Keywords:** adiposity, bisphenol, body mass index, endocrine disruptors, endocrine-disrupting chemicals, obesity, phthalate

## Abstract

Excess body weight constitutes one of the major health challenges for societies and healthcare systems worldwide. Besides the type of diet, calorie intake and the lack of physical exercise, recent data have highlighted a possible association between endocrine-disrupting chemicals (EDCs), such as bisphenol A, phthalates and their analogs, and obesity. EDCs represent a heterogeneous group of chemicals that may influence the hormonal regulation of body mass and adipose tissue morphology. Based on the available data from mechanistic, animal and epidemiological studies including meta-analyses, the weight of evidence points towards the contribution of EDCs to the development of obesity, associated disorders and obesity-related adipose tissue dysfunction by (1) impacting adipogenesis; (2) modulating epigenetic pathways during development, enhancing susceptibility to obesity; (3) influencing neuroendocrine signals responsible for appetite and satiety; (4) promoting a proinflammatory milieu in adipose tissue and inducing a state of chronic subclinical inflammation; (5) dysregulating gut microbiome and immune homeostasis; and (6) inducing dysfunction in thermogenic adipose tissue. Critical periods of exposure to obesogenic EDCs are the prenatal, neonatal, pubertal and reproductive periods. Interestingly, EDCs even at low doses may promote epigenetic transgenerational inheritance of adult obesity in subsequent generations. The aim of this review is to summarize the available evidence on the role of obesogenic EDCs, specifically BPA and phthalate plasticizers, in the development of obesity, taking into account in vitro, animal and epidemiologic studies; discuss mechanisms linking EDCs to obesity; analyze the effects of EDCs on obesity in critical chronic periods of exposure; and present interesting perspectives, challenges and preventive measures in this research area.

## 1. Introduction

Over recent decades, the escalating global increase in overweight and obesity, also known as “globesity”, has constituted one of the major health challenges for societies and healthcare systems worldwide. Based on the World Obesity Atlas 2023 report, approximately 38% of the world population presents with excess body weight, having a body mass index (BMI) of more than 25 kg/m^2^ [1]. This global prevalence of overweight and obesity is expected to reach 51% by 2035, while 78% of US adults are estimated to live with excess body weight by 2030 [2,3]. More striking than the elevated obesity rates in adults is the higher prevalence of excess body weight among children and adolescents, which has doubled or tripled in children of school age in many developed regions of the world [3,4]. Moreover, obesity is associated with a plethora of comorbidities, including diabetes mellitus type 2 (T2DM), coronary heart disease, hypertension, stroke, dyslipidemia, sleep apnea, osteoarthritis and some types of cancer [5,6,7,8].

During the past few decades, several research teams have investigated the association between common chemical exposures and the occurrence of allergy, asthma, immune dysfunction, cancer and other entities, including obesity [9,10]. The contemporary industrialized environment in developed countries is a constant source of a variety of chemical substances, presenting persistence and bioaccumulation potency in the food chain. Moreover, unrecognized or little-recognized environmental chemicals, such as industrial endocrine-disrupting chemicals (EDCs) or endocrine disruptors, have the potential to disrupt the actions of hormones, causing adverse health effects. Particularly, some EDCs that have been termed “obesogens” may also influence adipogenesis and regulatory metabolic pathways, leading to an imbalance in the regulation of body weight resulting in weight gain and obesity [11]. The most important EDCs that have been suspected in the development of obesity and obesity-associated metabolic disorders are the plastic additive bisphenol A (BPA) and the phthalate-based plasticizers. The aim of this narrative review is to summarize the available evidence on the role of EDCs, specifically BPA and phthalates, in the development of obesity, taking into account mechanistic, animal and epidemiologic studies; discuss mechanisms linking EDCs to obesity; analyze the effects of EDCs on obesity in critical chronic periods of exposure; and present interesting perspectives and challenges in the research area.

## 2. Methods of Literature Search and Review Criteria

Although this manuscript is not a systematic review, for its preparation, we applied the MESH search terms “obesity” and “endocrine-disrupting chemicals” in the PubMed NIH database from inception until 20 October 2023, which yielded 670 outputs. Among the 670 manuscripts, we excluded 19 manuscripts that were associated with brain and/or neurogenerative disorders, 14 manuscripts that were related to environmental factors only, 11 manuscripts that were referring to cancer, 4 articles related to rheumatic/bone diseases, 3 articles related to infectious diseases and 1 item that was a book and not an article. Furthermore, five manuscripts were written in French, two in German, one in Chinese and one in Japanese. Therefore, out of the 670 outputs, 61 were excluded for the aforementioned reasons, leaving a total of 609 manuscripts included in this literature search. Finally, we acknowledge that all these manuscripts cannot be covered in the context of this review.

## 3. Genetic and Environmental Factors in Obesity

Obesity is a multifactorial disorder characterized by abnormal or excessive fat accumulation that results from either an increase in the adipocyte size (hypertrophia) or an increase in the number of adipocytes (hyperplasia) and presents several risks to health. In clinical practice, BMI, which is closely associated with body fatness, is recommended by the WHO as a population-level measure of overweight and obesity, defined as a BMI of or above 25 kg/m^2^ and 30 kg/m^2^, respectively. [12,13,14]. Body fat percentage (BF%) as a % of total body weight presents some advantages over BMI in evaluating fat mass. The BF% cut-off points for obesity proposed by the WHO are 25% for men and 35% for women, corresponding to a BMI of 30 kg/m^2^ in young Caucasians [15].

Obesity represents a complex disorder due to a multifactorial etiology comprising genetic, epigenetic, environmental, societal and behavioral factors. Increased calorie diet, sedentary lifestyle and decreased energy expenditure play an important role [16,17]. Obesity has a strong genetic background demonstrated by family, twin and adoption studies that have shown heritability rates from 40 to 70% for BMI [18,19,20,21]. Nevertheless, non-syndromic monogenic obesity involves less than 5% of the obese population. More than 95% of subjects with obesity present common polygenic obesity, which is multifactorial and still poorly explained [22]. In particular, the relative contribution of polygenic scores from genome-wide association studies (GWASs) has been estimated to be between 2 and 8%, with the greater part of the BMI variation being unexplained [23]. The dominant challenge is a combination of genetic and environmental parameters such as physical exercise, energy intake and tobacco smoking, also including gene–gene and gene–environment interactions that underlie the complex and dynamic etiopathogenesis of obesity [24,25]. Furthermore, the accelerated global spread of excess body weight cannot be attributed to genetic factors only [26]. However, the availability of high-calorie food, the type of diet and physical inactivity are not sufficient to explain the striking increase in excess body weight. Therefore, environmental parameters may be responsible for the elevated rates of increased body weight during the last few decades [27]. Recent data have highlighted the role of other environmental factors contributing to the increase in BMI, such as EDCs.

## 4. The Spectrum of Endocrine Disruptors

Recent data from observational, animal and experimental studies have shown that certain chemical substances, such as EDCs, may have an impact on the endocrine system, being involved in the development and rapid propagation of obesity [28]. Based on the definition by the “Global assessment on the state of the science of endocrine disruptors” of the World Health Organization (WHO) in 2002, an endocrine disruptor has been described as “an exogenous substance or mixture that alters functions of the endocrine system and consequently causes adverse health effects in an intact organism, or its progeny, or (sub)populations” [29]. Certain EDCs are found in nature, such as phytoestrogens; however, the majority of EDCs are synthetic compounds that have been released through human activities into the ecosystem. Humans are exposed to endocrine disruptors through various sources in their daily lives, in outdoor and indoor environments and via the use of personal care and household products, industrial chemicals, pharmaceuticals, pesticides, herbicides, fungicides and flame retarders; air pollution; and dietary habits. EDCs constitute a highly heterogeneous group of chemical substances that can be categorized according to their chemical structure and properties, their occurrence and intended use, their mechanism of action, the potential direct or indirect impact on the endocrine system, the accumulation in the organism, their environmental persistence and the described or suggested clinical implications [30]. The catalog of EDCs encompasses agrochemicals, such as pesticides, herbicides and fungicides; industrial organic solvents/lubricants and their byproducts (dioxins, polychlorinated bisphenyls, polybrominated bisphenyls); pharmaceutical substances; catalysts; and plastic contaminants and plasticizers, such as bisphenols and phthalates [31]. In comparison to other EDCs, bisphenols and phthalates are metabolized and excreted relatively quickly (i.e., half-lives less than 24 h) and hence are considered nonpersistent [32]. Despite their nonpersistent nature, their ubiquitous and frequent use in a wide variety of consumer products throughout life leads to a chronic exposure, reported to be global [33].

**Figure 1:**
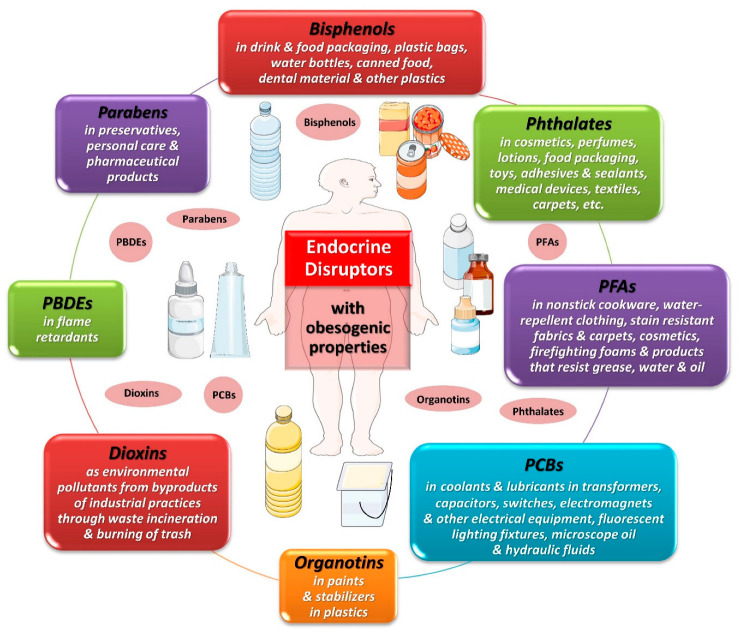
Obesogenic endocrine-disrupting chemicals. Abbreviations: PBDEs: polybrominated diphenyl ethers; PCBs: polychlorinated biphenyls; PFAs: perfluoroalkyl substances. All images are originated from the free medical site http://smart.servier.com/ (accessed on 1 December 2023) by Servier licensed under a Creative Commons Attribution 3.0 Unported License.

Potential targets of EDCs include any endocrine organ, hormonal system and/or hormonally affected pathway [34]. Some of these EDCs may interfere with the regulation of metabolism, energy balance and the storage of fat in the organism, leading to the development of obesity by affecting the function of adipose tissue and disrupting metabolic endocrine signaling [35]. Figure 1 depicts some EDCs that have been implicated in obesity. Some of the best-documented groups of EDCs with obesogenic properties and widespread exposure in the general population are bisphenols and phthalates, which are mainly found in plastic products.

**Figure 2:**
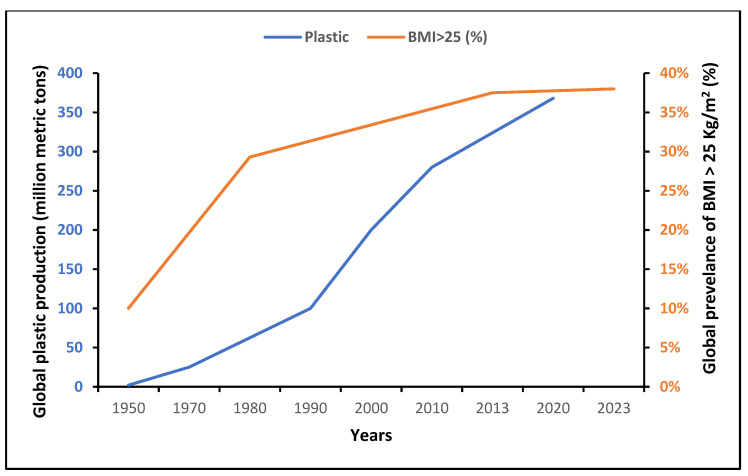
Worldwide plastic production in million metric tons and prevalence of excess body weight (BMI: >25 kg/m^2^) in percentage (%). Figure is based on data from [1,36,37].

Globally, the production of plastics has increased significantly since the mid-20th century (Figure 2). After a stagnation in 2020 due to the emergence of the COVID-19 pandemic, this production reached 390.7 million tons in 2021 [36]. Interestingly, there was a parallel striking epidemic rise in obesity/overweight globally, as depicted in Figure 2, mainly attributed to the international food production and supply system [3,37]. Since 1970, food production and supply with ameliorated manufacturing and distribution systems have radically shifted in the direction of elevated energy availability [3].

Worldwide, more people have access to palatable, cheap and ultra-processed foods of lower nutritional quality, and a number of obesogenic EDCs have gradually reached the global food chain, with a potential influence on human metabolism. Moreover, plasticizers are the main additives in the manufacture of plastic products, commonly used in the production of food packaging materials to improve flexibility, durability, processing and resistance to heat, fire and UV radiation. All these chemicals may be detached from polymer compounds and leak into the surrounding environment after being degraded. Humans may be exposed to those EDCs through various routes including food ingestion, the respiratory tract and dermal exposure [10].

### 4.1. Bisphenol A

**Figure 3:**
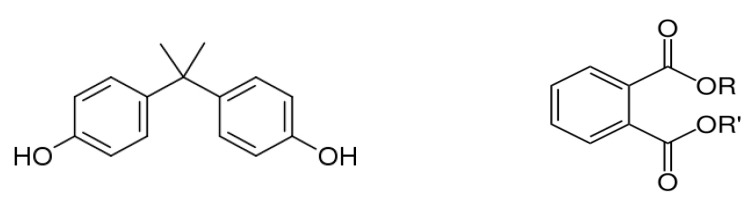
Chemical structure of bisphenol A (**left**) and phthalates (**right**).

Bisphenol A (BPA), or 4,4 -isopropylidenediphenol 2,2-bis (4-hydroxy-phenyl)- propane, was first reported by the chemist A.P. Dianin in 1891. It is a functional diphenyl compound that possesses two hydroxyl groups in the “para” position (Figure 3), which allows it to bind with androgen and estrogen receptors (classical nuclear ERα and ERβ, ERγ and membrane-associated GPR30) as an antagonist or agonist [38]. BPA is classified as a xenoestrogen due to its similarity with diethylstilbestrol, a synthetic estrogen, and as an EDC which enhances the ER with lower affinity compared to 17β-estradiol [39,40].

Due to its elastic, cross-linking, polymer-forming and intrinsic heat resistance properties, BPA represents one of the world’s most heavily produced synthetic industrial chemical compounds listed by the Organization for Economic Cooperation and Development, with more than 15 billion pounds produced worldwide annually [41,42,43] and over 1 million pounds leached into the environment [44]. BPA is used worldwide in the synthesis of polycarbonate plastics, plastic consumer products, drink and food packaging, plastic bags, water bottles, epoxy resin linings of beverage containers and canned food, dental materials such as sealants, electronic equipment, toys, optical lenses, paper coatings, adhesives, dye developers and thermal papers [45].

BPA can be released into the surrounding environment through various means including exposure to heat or acidic conditions, hydrolysis or degradation of the polymer and constant diffusion of residual BPA that remains on the polymer [46]. The quantity of released BPA is affected by duration of exposure, processing methodology and environmental conditions such as temperature and PH [47]. Human exposure to nanomolar concentrations of environmental BPA is continuous and widespread via oral, skin and respiratory absorption from atmospheric exposure, and dust particles from commercial and residential environments [48]. Exposure to BPA in utero and through lactation is also important for the developing fetus and the neonate, respectively [49,50]. However, the main route of exposure originates from water and diet, as BPA may migrate from beverage and food packaging, as well as from dental sealants [46,51]. Interestingly, skin exposure is more severe than oral intake due to the sustained presence of BPA in the organism and the elevated plasma concentrations of unconjugated toxic BPA [52]. However, this kind of exposure does not reflect a natural setting. Moreover, Christensen et al. reported that the majority of BPA exposure was food-related (i.e., via leaching from food packaging materials and containers) after a fasting study to exclude food intake as a potential route of BPA exposure [53].

BPA and its conjugates have been detected in various body fluids and tissues, including urine, saliva, plasma, feces, amniotic fluid and breast milk; however, urine samples are mainly examined for human monitoring. Epidemiologic studies have confirmed the widespread exposure to BPA with 95 to 99.8% of adults, adolescents, children and infants presenting detectable concentrations of BPA and its metabolites in urine, independently of gender, income, educational level or BMI [54,55]. Additionally, circulating free unconjugated BPA, which is the active form of BPA, is usually determined at concentrations of nanograms per millimeter in serum or plasma [56]. BPA kinetics analyses have shown that the rate of excretion of BPA is not highly influenced by fasting, suggesting a slower rate of BPA excretion or a potential bioaccumulation of BPA in human tissues, particularly in adipose tissue [45,49]. Interestingly, in a study of the urinary BPA profile in five individuals over a 48-h period of fasting (bottled water only), BPA levels increased after the pre-fast meal, decreased over the next 24 h, fluctuated at lower levels during the second day, and then rose after the post-fast meal [53]. This rise may be attributed to non-food sources that could be still present, such as dust, or the release of BPA from lipid reservoirs from past exposures [53].

Exposure to BPA has been associated with a plethora of disorders such as obesity, T2DM, cardiovascular disease, infertility, neurodegenerative diseases and cancer, particularly breast cancer [57]. Moreover, BPA is classified as a potentially toxic and harmful substance to reproduction and eyes, respectively, and a possibly irritating substance to the skin and respiratory tract [58].

Due to increasing concerns about the safety of BPA and evidence of its relationship with human health, BPA has been banned in the manufacture of baby bottles since 2011 in the European Union (EU). The U.S. Food and Drug Administration (FDA) banned the use of BPA in baby bottles, spill-proof cups and infant formula packaging materials in 2012–2013. In April 2023, the European Food Safety Authority (EFSA) published a re-evaluation of BPA’s safety, drastically decreasing the tolerable daily intake (TDI) for BPA from 4 μg/kg of body weight per day in 2015 to 0.2 ng/kg (around 20,000 times lower than before) [59]. These restrictions were mainly based on toxicological animal studies that appraised BPA side effects on renal, hepatic and immune function [46,60]. Nevertheless, since there is an absence of international standardization regarding a tolerable BPA limit, BPA is actually used in the production of polycarbonate plastic food materials. Following the bans and restrictions, the industry has gradually employed a variety of less-studied BPA analogs with toxicological characteristics that are not fully elucidated. However, like BPA, alternatives such as bisphenols S (BPS), F (BPF), B (BPB), C (BPC), E (BPE), AF (BPAF), P (BPP) and Z (BPZ) and 4-cumylphenol (HPP) have also been identified as EDCs, presenting androgenic, estrogenic and obesogenic activity in vitro as well as neurotoxic, genotoxic and cytotoxic potential [46,57,60,61,62,63,64]. BPA analogs may bind to nuclear receptors due to their phenyl moiety and hydrophobic structure that play a role in endocrine-disrupting activity [65].

### 4.2. Phthalates

Phthalates are a group of chemical compounds in the family of esters derived from phthalic acid. The common chemical structure of phthalates involves a benzene ring with two carboxylic acid groups (ortho-phthalates, Figure 3) or one carboxylic acid group (para-phthalates) attached. The length and structure of the alkyl side chains attached to these carboxylic acid groups vary, resulting in different types of phthalates with specific properties [66]. Ortho-phthalates represent the most common type of phthalates and include diethyl phthalate (DEP), dibutyl phthalate (DBP), diisobutyl phthalate (DIBP), diisononyl phthalate (DINP) and di(2-ethylhexyl) phthalate (DEHP). Phthalates first appeared in the 1920s, and their widespread use as plasticizers destined primarily to soften polyvinyl chloride (PVC) started in the 1930s. Currently, the total production of phthalates is approximately 5.5 million tons per year with an increasing rise attributed to the use of PVC [67]. Phthalates constitute a large fraction of polymer additives due to the presence of various beneficial properties, such as flexibility, compatibility with polymers, chemical stability and durability, low water solubility, resistance to heat and weather conditions, electrical resistivity and transparency. They are primarily used as plasticizers in the production of flexible PVC products (vinyl flooring, PVC cables and wires, etc.); personal care products in cosmetics, perfumes and lotions to enhance fragrance and texture; toys and children’s products; adhesives and sealants; medical devices; building materials; coatings and inks; textiles and carpets; and food packaging [67,68].

Because phthalates are not covalently bound to their compounds, they may easily migrate and leak into the environment, where they can accumulate in the food chain due to their lipophilic nature [69]. Hence, humans are exposed to phthalates through dietary exposure, which represents the major route for intake; skin absorption; and inhalation of indoor and outdoor air comprising dust [30,70]. Infants, toddlers and children have a higher exposure to phthalate esters than adults through their mouthing activity and contact with toys, carpets and floors [30]. Furthermore, certain phthalates have the ability to pass through the placenta, potentially exposing the developing fetus to these chemicals during pregnancy [71,72]. Epidemiological studies have shown that phthalates, particularly the commonly used DEHP and its breakdown products, may be found in a plethora of human body fluids such as urine, which is the most commonly studied and used matrix for assessing human exposure to phthalates; plasma; saliva; follicular fluid; amniotic fluid; and breast milk [72,73,74,75]. Phthalate esters present endocrine-disrupting properties associated with detrimental reproductive and neurodevelopmental effects as well as obesity and T2DM [30,76,77,78]. In response to concerns about the potential harmful effects of phthalates, the use of certain phthalates including DEHP has been restricted in concentrations exceeding 0.1% by weight of the plasticized products in Europe and elsewhere [79]; nevertheless, there are no international acceptable limits for phthalate esters. Efforts have been made to reduce or replace their use in various applications with non-phthalate and bio-based plasticizers, polymer blends and formulations, and alternative materials. Interestingly, exposure to most EDCs, including DEHP metabolites and BPA, decreased between 2009 and 2016 in a sample of individuals with impaired fasting glucose from the Dutch population [80]. However, there is a need to assess the use of less toxic substitute chemical compounds for their metabolic and endocrine consequences.

## 5. Mechanisms Linking Endocrine Disruptors to Obesity

The hormonal system plays a crucial role in the regulation of carbohydrate, lipid and protein metabolism as well as in the regulation of body mass. Moreover, adipose tissue is not considered a passive storage depot for energy; it is also recognized as a large endocrine organ secreting a plethora of various hormones and bioactive molecules (adipokines) that play a critical role in regulating metabolism and body mass as well as other functions [81]. EDCs are xenobiotics exhibiting endocrine disruptor properties by binding to various nuclear receptors, altering specific cellular responses. These actions are pleiotropic depending on the cell and receptor types, receptor density, critical chronic periods of exposure, the presence of other cofactors, etc. Some important properties of EDCs include the following: (1) their effects at very low concentrations in a similar fashion to hormonal effects; (2) the exhibition of U-shaped nonmonotonic responses where decreased levels may cause phenotypic alterations not necessarily seen with increased doses; (3) agonistic or antagonistic actions after interacting with hormonal receptors resulting in complex unpredictable biological responses with stimulation, mimicking or inhibition of hormonal effects; (4) their impact on the number of cellular receptors and the level of serum hormones; and (5) their accumulation in adipose tissue due to their lipophilic nature which further increases the retention of other lipophilic chemicals [82].

**Figure 4:**
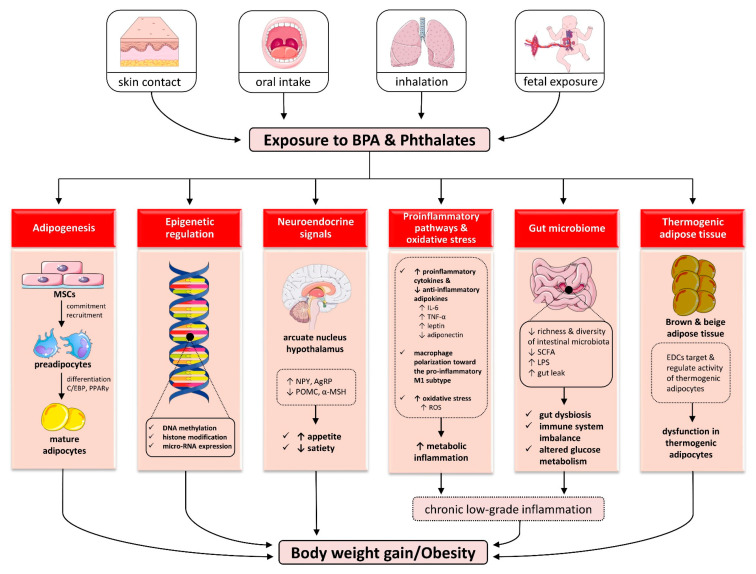
Main mechanisms linking endocrine-disrupting chemicals such as BPA and phthalates to obesity. Abbreviations: AgRP: Agouti-related peptide; α-MSH: α-melanocyte-stimulating hormone; BPA: bisphenol A; C/EBP: CCAAT/enhancer-binding family of proteins; EDCs: endocrine-disrupting chemicals; IL: interleukin; LPS: lipopolysaccharide; MSCs: mesenchymal stem cells; NPY: neuropeptide Y; POMC: proopiomelanocortin; ROS: reactive oxygen species; SCFA: small-chain fatty acid; TNF-α: tumor necrosis factor-α. All images are originated from the free medical site http://smart.servier.com/ (accessed on 1 December 2023) by Servier licensed under a Creative Commons Attribution 3.0 Unported License.

BPA and phthalates may interact with several nuclear hormone receptors including ERα and ERβ, with an affinity being 10^3^–10^4^ times lower compared to 17β-estradiol (E2), androgen receptor (AR), progesterone receptor (PR), glucocorticoid receptor (GR), thyroid hormone receptors (TRα and TRβ), G-protein-coupled receptor 30 (GPR30), estrogen-related γ receptor (ERR-γ), mineralocorticoid receptor (MR), peroxisome-proliferator-activated receptors (PPARs) and retinoid receptors [83,84,85,86,87]. Obesogenic EDCs such as BPA and phthalates may be implicated in the etiopathogenesis of obesity and associated metabolic disorders by (1) increasing the number and size of adipocytes through the regulation of genes involved in adipogenesis; (2) modulating epigenetic pathways during development, enhancing susceptibility to obesity; (3) impacting neuroendocrine signals responsible for appetite and satiety; (4) promoting a proinflammatory milieu in adipose tissue and inducing a state of chronic subclinical inflammation; (5) dysregulating gut microbiome and immune homeostasis; and (6) inducing dysfunction in thermogenic adipose tissues. Figure 4 depicts the main mechanisms linking EDCs to excess body weight.

Based on animal and epidemiological studies, critical periods of exposure to obesogenic EDCs are in utero and the neonatal period. Moreover, EDCs can promote epigenetic transgenerational inheritance of obesity in adulthood in subsequent generations [88]. Fetal development is sensitive to maternal exposure to infectious agents, alcohol, drugs and toxins including EDCs [89]. The majority of EDCs cross the placenta barrier through active or passive transportation and may be found in the amniotic fluid or the placenta. Another critical period for exposure to EDCs is puberty due to the activation of the hypothalamic–pituitary–adrenal and gonadal axes. Certain animal studies have shown precocity or delay of puberty after exposure to EDCs with implications in body mass [89].

### 5.1. Effects on Adipogenesis

Obesity is characterized by the expansion of fat tissue, known as adipogenesis, attributed to hyperplasia via the differentiation of resident precursors or their increase in size (hypertrophy). Current data have shown that the number of adipocytes is set by the end of childhood, constituting a major predictor of fat mass in adulthood [90]. EDCs may influence both the linear commitment from mesenchymal stem cells (MSCs) to pre-adipocytes and the differentiation of pre-adipocytes into mature adipocytes, regulated by several adipogenic transcription factors such as the group of CCAAT/enhancer-binding family of proteins (C/EBPα, β, δ) that stimulate the nuclear [35] receptor PPAR-γ which is a critical regulator of adipocyte differentiation [91].

Mechanistic studies performed in 3T3-L1 cells (type of mouse fibroblast-like cell line that can differentiate into adipocytes), human adipose-derived stem/stromal cells and uncommitted NIH/3T3 cells have shown that exposure to BPA and its analogs upregulates the expression of C/EBPα and PPARγ, inducing adipocyte differentiation and lipid accumulation [61,92,93,94]. Although some phthalates and related metabolites exhibit greater effects on PPARα, particularly in mice, they can be potent and selective activators of PPARγ promoting adipogenesis [86,95,96,97]. Additive effects of a mixture of EDCs on adipogenesis may also be observed [35]. Interestingly, exposure to environmentally relevant doses of BPA, phthalates or their analogs in early life may result in elevated body weight and fat tissue mass in male and female mice by altering the recruitment and differentiation of adipocytes [98,99]. Finally, in human studies, an association between higher urinary BPA levels and obesity, metabolic syndrome, T2DM and cardiovascular disease has been reported in the general adult US population [100,101].

The exact molecular mechanism through which BPA and phthalate induce adipocyte differentiation is unclear; however, many mechanisms are unraveled and synopsized in Section 6 and Section 7.

### 5.2. Effects on Epigenetic Regulation

Out of the many potential mechanisms modulating gene expression in adipose tissue, epigenetic alterations have been of particular interest in the last few years. Epigenetics is defined as alterations in gene function, such as DNA methylation, histone modifications and microRNA (miRNA) interference, that occur without any modification in the DNA sequence.

EDCs may affect epigenetic pathways during early development and childhood, resulting in epigenetic alterations and predisposition to obesity. The interconnection of obesogenic EDCs and epigenetic alterations involves DNA methylation affecting the activity of DNA methyltransferases and their cofactors such as methyl donor S-adenosylmethionine or the modulation of locus-specific epigenetic patterns, histone modifications and miRNA expression, with most data focusing on DNA methylation [88].

BPA was shown to reduce global DNA methylation and activate adipocyte differentiation in 3T3-L1 cells [102]. Thus, altered epigenetic gene regulation could play a role in the association between BPA exposure and obesity development. In another study, prolonged low-dose BPA exposure in committed 3T3L1 and uncommitted NIH3T3 pre-adipocytes showed that its effects on adipogenesis are mediated through epigenetic mechanisms via a reduction in the PPAR-γ promoter methylation in pre-adipocytes mainly [103]. Interestingly, the ending of the BPA exposure may reverse the PPAR-γ promoter methylation and inflammatory pattern of the 3T3L1 cells [103]. Therefore, preventing BPA exposure is important for metabolic health. Moreover, butyl benzyl phthalate (BBP) may induce differentiation of 3T3-L1 cells and C3H10T1/2 stem cell lines, with increased expression levels of miR-34a-5p. MiR-34a-5p is involved in adipogenesis, obesity and potentially the epigenetic regulation of insulin signaling [104,105].

Developmental BPA exposure may modify and reprogram the liver β-oxidation function in male rats via the epigenetic regulation of genes (DNA methylation and histone modifications) implicated in β-oxidation, such as the carnitine palmitoyltransferase (*Cpt1α)* gene [106]. BPA changed DNA methylation and histone marks (H3Ac, H4Ac, H3Me2K4, H3Me3K36) and reduced the binding of several transcription factors (Pol II, C/EBPβ, SREBP1) in the *Cpt1a* gene, which is critical for β-oxidation [106]. Hence, BPA toxicity is characterized by DNA methylation and histone modifications.

Exposure to phthalate and its metabolites in utero may affect DNA methylation, leading to long-term implications in body weight [88]. Prenatal exposure to DEHP may impact fetal development [107]. A cord blood epigenome-wide study identified elevated methylation changes in genes associated with metabolism, the endocrine system and signaling pathways that were positively linked to maternal blood mono-(2-ethylhexyl) phthalate (MEHP) levels [107].

Larger and longitudinal multi-omics studies are needed to elucidate the effects of prenatal exposure to EDCs on fetal growth and long-term metabolic outcomes.

### 5.3. Effects on Neuroendocrine Signals of Appetite and Satiety

Obesogenic EDCs may influence the neuroendocrine control of appetite, satiety and food preference. Bisphenols, including BPA, have been detected in post-mortem human hypothalamic and white matter brain material [108]. In this study, the majority of bisphenol concentrations were associated with obesity. The accumulation of bisphenols in white-matter-enriched brain tissue may underscore their ability to cross the blood–brain barrier [108].

Exposure to BPA in utero and in adulthood may cause the arcuate nucleus of the hypothalamus to generate elevated levels of orexigenic neuropeptides such as neuropeptide Y (NPY) and Agouti-related peptide (AgRP), resulting in an increase in appetite [109,110,111].

Interestingly, NPY can also enhance lipogenic enzymes in fat tissues, leading to the development of obesity [112]. On the contrary, proopiomelanocortin (POMC), which generates the anorexigenic neuropeptide α-melanocyte-stimulating hormone (α-MSH), is diminished [109]. Moreover, exposure to BPA and its analog tetrabromobisphenol A (TBBPA) at environmental levels resulted in hyperphagia and obesity in adult male zebrafish by stimulating the endocannabinoid receptor type 1 (CB1), which plays an important role in the gut–brain axis control of appetite and satiety [113]. Phthalates such as DEHP may upregulate the expression levels of NPY in male rats [114]. Finally, in a prospective epidemiological study, BPA was strongly linked to the orexigenic gut hormones ghrelin and leptin particularly in women, suggesting that BPA could be implicated in the hormonal control of appetite and satiety [115].

### 5.4. Effects on Proinflammatory Pathways and Oxidative Stress

BPA and phthalates may induce the expression and secretion of certain proinflammatory cytokines such as interleukin (IL)-6 and tumor necrosis factor (TNF)-α and downregulate the expression of anti-inflammatory adipokines such as adiponectin, leading to a sustained low-grade inflammation both locally and systematically, adipose dysfunction and insulin resistance [84,116,117]. Nevertheless, the mechanisms implicated in metabolic inflammation are still under investigation. The majority of in vitro studies in 3T3-L1 cells and in adipocytes derived from subcutaneous tissues have shown that exposure to 1 nM BPA induced an increase in IL-6 and interferon (IFN)-γ while prolonged exposure upregulated the mRNA levels of IL-6, IL-1β, IFN-γ and monocyte chemoattractant protein-1 (MCP1), which were reversed after BPA removal [84,118]. These results were confirmed in some animal studies; however, the effect of BPA depends on caloric intake, diet composition and gender [84]. Furthermore, BPA and its substitutes could promote macrophage polarization toward the proinflammatory M1 subtype, which may be reversed by ERα inhibition in the case of BPF [119]. BPA and certain phthalates may also enhance the generation of reactive oxygen species (ROS), which is crucial in cellular stress and the progression of inflammation [120,121]. Finally, in many epidemiological studies, urinary and plasma BPA correlated positively with somatometric indices, such as BMI and waist circumference (WC); proinflammatory biomarkers, including IL-17, a cytokine implicated in chronic inflammation; and glycemic indices (insulin and glucose) [84,122,123,124].

### 5.5. Effects on Gut Microbiome

BPA and phthalates that accumulate in the gut may influence the gut microbiome by altering its composition, resulting in gut dysbiosis, immune system imbalance and altered glucose metabolism [60]. Exposure to BPA during gestation may promote obesity phenotypes in murine models by altering gut microbiota [109]. BPA exposure decreases the richness and diversity of the intestinal microbiota and beneficial small-chain fatty acid (SCFAs), induces leaky gut and elevates systemic lipopolysaccharide (LPS), resulting in chronic subclinical inflammation and altered lipid and glucose homeostasis [125,126]. In a mouse multi-omics study, prenatal exposure to low-dose BPA altered the expression of hepatic genes implicated in oxidative phosphorylation, fatty acid metabolism and PPAR signaling and impacted intestinal bacterial diversity in an age- and gender-dependent fashion [127]. Exposure to phthalate esters such as DEHP elevated cardiovascular risk in obese mice by dysregulating the arachidonic acid metabolism of intestinal flora [128], while prolonged exposure increased weight and liver lipogenesis in mice by inducing the uptake of fatty acids and dysregulating the metabolism of phospholipids and choline [129].

### 5.6. Effects on Thermogenic Adipose Tissue

Certain natural and synthetic EDCs, including BPA and phthalates, may induce dysfunction not only in white adipocytes but also in thermogenic brown and beige adipocytes, which regulate thermogenesis, fat metabolism and energy balance [130]. Brown and beige adipocytes share common features that comprise the existence of multiple dense mitochondria, lipid droplets and the expression of uncoupling protein 1 (UCP1) which permits the production of heat at the expense of ATP production through oxidative phosphorylation [131]. Therefore, these cells can prevent hypothermia without shivering and metabolize excess fat via UCP1.

Although data on the effects of EDCs on thermogenic adipose tissue are scant, growing evidence suggests that bisphenols and phthalates may target and regulate the activity of thermogenic adipocytes. BPA and DEHP preferentially accumulate in mice thermogenic adipose tissues at greater levels than those determined in serum, white adipose tissue (WAT) and the brain [132,133]. Nevertheless, their effects depend on the animal used, gender, life stage, type of exposure, and concentrations of specific chemical compounds. For example, there is a sexual dimorphism observed after gestational exposure to BPA with increased brown adipose tissue (BAT) depots and UCP1 in female offspring and decreased BAT activity and adipogenesis in male offspring [134]. The same applies to developmental exposure to DEHP where a decrease in BAT activity accompanied by hypothermia, hyperphagia and weight gain was observed in male mice [135] and hyperplastic brown fat was seen in female offspring [133]. These actions may be attributed to agonistic or antagonistic effects of EDCs on ERs, the impaired thyroid signaling that impacts the beiging of WAT and the macrophage subpopulation located in the WAT [130,136]. Finally, differential effects of bisphenols and phthalates are observed in macrophage polarization promoting an environment permissive for the beiging of WAT [130]. For example, BPA and BPF induce classical macrophage M1 polarization with increased secretion of proinflammatory cytokines that affect adipocyte beiging while DEHP may promote macrophage M2 polarization facilitating beiging [130]. More studies are needed to delineate the effects of EDCs on thermogenic adipose tissues, which are critical in the regulation of energy balance.

## 6. Evidence from Mechanistic Studies Linking BPA and Phthalates to Obesity

Existing experimental evidence has shown that BPA and phthalates may exert their obesogenic properties by promoting the differentiation of pre-adipocytes or MSC stems into mature adipocytes (Table 1). In addition, these endocrine disruptors may predispose individuals to obesity through metabolic alterations in mature adipocytes. Prolonged BPA exposure at low, environmentally relevant, concentrations may induce pre-adipocyte proliferation and differentiation due to the increased expression of adipogenic transcriptional factors, including PPARγ. It is worth noting that these effects of BPA exposure are relevant to the environmental concentrations, resulting in adipocyte metabolic dysfunction and proinflammatory cytokine production [137]. Moreover, MEHP exposure at the dose of 10 µM activates undifferentiated pre-adipocytes, leading to de novo lipogenesis, through the augmented expression of glucose transporter (GLUT)1, GLUT4 and S100B [138]. Interestingly, BPA and DEHP may promote the differentiation of murine MSCs into adipocytes at different concentrations and stages of cell determination and differentiation [139]. Furthermore, BBP potentially impairs pre-adipocyte differentiation through epigenetic changes of genes implicated in adipogenesis since BBP exposure augments miR-34a-5p expression and attenuates the expression level of its target genes [104].

On the other hand, low BPA doses may provoke adipose tissue dysfunction, without markedly interfering with adipocyte differentiation or the activation of adipogenic factors. In these cases, adipogenesis is suggested to be mediated by inflammatory and insulin signaling pathways. Alternatively, the activation of specific cellular receptors has been proposed. Thus, BPA and 2,4-dichlorophenol (DCP) may promote pre-adipocyte differentiation in the 3T3-L1 cells, through activating GRs, without increasing PPARγ expression [85]. In addition, the increased expression of insulin-like growth factor 1 (IGF-1) under BPA exposure may enhance adipogenesis via ER stimulation [140]. In mature adipocytes, DINP and DPHP exposure may lead to insulin resistance and inflammation as a result of oxidative stress, mitochondrial dysfunction and augmented adipokine secretion [141]. In turn, BPA may diminish insulin-stimulated glucose utilization. Moreover, exposure to BPA may stimulate intracellular signaling pathways that promote apoptosis (c-Jun N-terminal kinases/JNKs) or activate transcriptional factors that induce the expression of proinflammatory cytokines (NF-kB) [118]. Interestingly, daily BPA exposure has been reported to increase insulin secretion in response to glucose stimulation in pancreatic cells via ERs, in contrast to acute (60 min) exposure, which has no impact [142]. De Filippis et al. have shown that BPA exposure had no impact on the expression of PPARγ, fatty-acid-binding protein 4 (FABP4) and fatty acid synthase (FASN) and pre-adipocyte differentiation, highlighting the implication of the increased expression of IL-6 and TNF-α [143]. Notably, BPA may induce adipogenesis through mTOR signaling and TR/RXR stimulation [144].

Multiple in vitro studies have indicated that exposure to BPA and phthalates may disrupt cellular lipid homeostasis through a variety of underlying mechanisms, including the regulation of specific signaling pathways. Thus, MEHP may induce lipid accumulation by inhibiting the Janus kinase (JAK)2/signal transducer and activator of transcription (STAT)5 signaling [145]. In addition, in vitro evidence in hepatic cellular models supports that BPA dysregulates lipid homeostasis through the enhanced expression of apolipoprotein A4 (APOA4) or via the activation of the CB1 receptor [146,147]. BPA in the lowest doses may promote the expression of the mRNA level of the 11β-HSD1 gene, resulting in adipogenesis and lipid synthesis [148]. In addition, BPA seems to be involved in the upregulation of several genes related to lipid metabolism, like lipoprotein lipase (LPL) and SREBF1. As shown by Grasselli et al., non-cytotoxic levels of BPA reduced the expression of genes involved in lipid oxidation but had no impact on the expression of lipogenic genes [149]. Furthermore, BPA and DEHP exposure may alter the expression of lipid metabolism markers, consequently resulting in steatosis in RTL-W1 cells [150]. Five years ago, Schaedlich et al. reported that DEHP exposure attenuates triacylglycerol (TG) accumulation in lipid droplets of human Simpson–Golabi–Behmel syndrome pre-adipocyte cells (SGBSs), in which reduced adiponectin levels and increased leptin levels were present [151].

In conclusion, EDCs may exert in vitro obesogenic effects through a plethora of mechanisms involving the disruption of adipocyte differentiation and inflammation as well as changes in insulin metabolism and sensitivity. Although they are of clinical importance, further studies reflecting obesogenic features in real life are needed to elucidate their potential association with obesity.

**Table 1:** Major in vitro studies portraying associations between BPA and phthalates, and obesity.

Authors, Year	Type of Cell Culture	Main Findings	Remarks
Bisphenol A and obesity			
Riu et al., 2011 [152]	NIH3T3-L1 cell line (pre-adipocytes)	1. ↑ adipogenesis 2. ↑ lipid accumulation 3. ↑ mRNA level of PPARγ 4. ↑ PPARγ activity	1. Animal in vitro model 2. ED: TBBPA 3. Obesogenic effects at 10 µM
Valentino et al., 2013 [118]	Primary hADSCs	1. (-) mRNA level of PPARγ, GLUT4 2. ↓ of glucose utilization 3. ↓ tyrosine phosphorylation of insulin receptor (IR) 3. ↓ of PKB/Akt phosphorylation 4. ↑ of IL-6, IFN-γ 5. ↑ of JAK/STAT, JNK 6. ↑ activity NF-kB pathway	1. Human in vitro model 2. ED: BPA 3. Biological effects at 1 nM
Bastos Sales et al., 2013 [102]	Murine N2A, human SK-N-AS neuroblastoma cells and murine pre-adipocyte fibroblasts (3T3-L1)	1. Modest ↓ in global DNA methylation in murine N2A cells 2. No changes in global DNA methylation in human SK-N-AS cells. 3. ↑ adipocyte differentiation in murine 3T3-L1 pre-adipocytes	1. Animal and human in vitro model 2. ED: BPA and a range of several EDCs not belonging to bisphenols 3. Biological effects at ≥ 10 μΜ
Menale et al., 2015 [137]	Primary pre-adipocytes	1. ↑ adipogenesis 2. ↑ lipid accumulation 3. ↑ mRNA level of ERα (10 nM, 100 nM) 4. (-) mRNA level of ERβ 5. ↑ production of IL1B, IL18, CCL20 (10 nM)	1. Human in vitro model 2. ED: BPA 3. Obesogenic effects at 1 nM, 10 nM, 100 nM
Ariemma et al., 2016 [92]	3T3-L1 Pre-adipocytes	1. Undifferentiated cells: - ↑ proliferation - ↑ differentiation - ↑ expression of PPARγ, C/EBPα and FABP4/AP2 2. Mature adipocytes: - Hypertrophy - ↑ lipid accumulation - ↑ mRNA of leptin, IL6, IFNγ - ↓ glucose utilization	1. Animal in vitro model 2. ED: BPA 3. Obesogenic effects at 1 nM
Longo et al., 2020 [103]	3T3L1 and NIH3T3 (committed and uncommitted pre-adipocytes, respectively)	- ↓ DNA methylation at PPARγ promoter, without affecting mRNA expression in pre-adipocytes - Transient ↑ in PPARγ expression and lipid accumulation at D4 of differentiation in 3T3L1 cells - Ending BPA exposure restores the PPARγ promoter methylation and inflammatory profile of 3T3L1 cells. - Expression of PPARγ is barely detectable and its promoter is completely methylated in NIH3T3 cells - ↑ PPARγ expression is more evident both in pre-adipocytes and during the adipocyte differentiation	1. Animal in vitro model 2. ED: BPA 3. Biological effects at low doses: 1 nM
Cohen et al., 2021 [153]	Primary hADSCs	1. ↑ adipogenesis and lipid production at 0.1 nM 2. ↓ adipogenesis and lipid production at 10 nM	1. Human in vitro model 2. ED: BPA 3. Biological effects at 0.1 nM, 10 nM
Yamasaki et al., 2021 [154]	ST-13 cell line (pre-adipocytes)	- Undifferentiated cells: 1. (-) lipid accumulation 2. (-) mRNA level of PPARγ 3. ↑ mRNA level of AACS, PLIN1, FAS, CIDEA, LSD-1 - Mature adipocytes: 1. (-) lipid accumulation 2. (-) mRNA level of AACS, SCOT	1. Animal in vitro model 2. ED: TBBPA 3. Obesogenic effects at 0.5 µM, 1 µM
Schaffert et al., 2021 [121]	SGBSs (pre-adipocytes)	1. ↑ binding to PPARγ (50 µM) 2. (-) PPARγ activity (10 nM, 100 nM, 1 µM, 10 µM) 3. ↓ lipid accumulation (10 nM, 100 nM, 1 µM, 10 µM) 4. ↑ leptin (10 nM) 5. ↓ cellular ROS level (10 nM, 100 nM, 1 µM, 10 µM) 6. ↓ insulin sensitivity (1 µM)	1. Human in vitro model 2. ED: BPA 3. Obesogenic effects at 10 nM, 100 nM, 1 µM, 10 µM, 50 µM
Marqueno et al., 2021 [155]	ZFL cell line (primary mouse hepatocytes)	1. ↑ lipid accumulation (5 µM, 50 µM) 2. ↑ ROS generation (20 µM, 50 µM, 70 µM, 100 µM, 150 µM, 200 µM)	1. Animal in vitro model 2. ED: BPA 3. Biological effects at 5 µM, 20 µM, 50 µM, 70 µM, 100 µM, 150 µM, 200 µM
Lee et al., 2022 [156]	Huh-7 cell line (primary hepatocytes)	1. ↓ cell viability (200 µM, 400 µM) 2. ↑ lipid accumulation (10 µM, 50 µM, 100 µM, 200 µM) 3. Fatty acid uptake ↑ (10 µM, 50 µM, 100 µM) 4. ↑ intracellular ROS formation (10 µM, 50 µM, 100 µM, 200 µM)	1. Human in vitro model 2. ED: BPA 3. Biological effects at 10 µM, 50 µM, 100 µM, 200 µM, 400 µM
Phthalates and obesity			
Sargis et al., 2010 [85]	3T3-L1 cell line (pre-adipocytes)	1. ↑ adipogenic differentiation 2. ↑ lipid accumulation (100 nM) 3. ↑ PPARγ and glucocorticoid-like activity (1 µM) 4. ↑ adiponectin and protein expression of IR-β (1 µM–100 pM)	1. Animal in vitro model 2. Pthalate: DCHP 3. Obesogenic effects at 100 pM, 1 nM, 10 nM, 100 nM, 1 µM
Dimastrogiovanni et al., 2015 [150]	RTL-W1 cell line (hepatocytes)	1. ↑ lipid accumulation 2. ↓ alteration of membrane lipids 3. ↓ mRNA level of CD36, FAS, LPL	1. Animal in vitro model 2. Pthalate: DEHP 3. Biological effects at 5 μΜ
Zhang et al., 2017 [105]	C3H10T1/2 cell line (MSCs)	1. ↑ adipogenesis 2. ↑ mRNA level of AP2, PPARγ 3. ↑ lipid accumulation 4. ↑ protein level of FOXO1 5. ↑ acetylation of FOXO1, β-catenin 6. ↓ protein level of SIRT1, SIRT3	1. Animal in vitro model 2. Pthalate: BBP 3. Biological effects at 50 μΜ
Schaedlich et al., 2018 [151]	SGBSs (pre-adipocytes)	1. ↓ TGsaccumulation 2. ↓ adiponectin production 3. ↓ protein level of PPARα, PPARγ 4. ↓ phosphorylation of ERK1, ERK2 5. ↑ lipolysis 6. ↑ ROS formation	1. Human in vitro model 2. Pthalate: DEHP 3. Obesogenic effects at 50 µg/mL
Zhang et al., 2019 [145]	BRL-3A cell line (hepatocytes)	1. ↑ lipid accumulation (100 µM, 200 µM) 2. ↑ mRNA level of FAS, PDK4, AP2 (10 µM, 50 µM, 100 µM, 200 µM) 3. ↑ mRNA level of PPARγ (50 µM, 100 µM, 200 µM) 4. ↓ JAK2/STAT5 signaling 5. ↓ level of indicators of oxidative stress: SOD ↓, MDA ↑ (10 µM, 50 µM, 100 µM, 200 µM)	1. Animal in vitro model 2. Pthalate: MEHP 3. Biological effects at 10 µM, 50 µM, 100 µM, 200 µM
Perez-Albaladejo et al., 2021 [157]	PLHC-1 cell line (hepatocytes)	- DBP: 1. ↑ TG accumulation (20 µM) 2. ↑ ROS formation (5 µM, 20 µM, 50 µM, 100 µM) - DEHP: 1. ↑ TG accumulation (5 µM, 10 µM) 2. ↑ ROS formation (100 µM)	1. Animal in vitro model 2. Phthalates: DBP and DEHP 3. Biological effects at - DBP: 5 µM, 20 µM, 50 µM, 100 µM - DEHP: 5 µM, 10 µM, 100 µM
Meruvu et al., 2021 [104]	3T3-L1 cells	- ↑ miR-34a-5p expression - ↑ adipogenesis - ↓ Nampt, Sirt1 and Sirt3 gene expression levels; ↓ Nampt protein - ↓ adipogenesis, ↑ Nampt protein and NAD+ after miR-34a-5p knockdown in the presence of BBP	1. Animal in vitro model 2. Phthalate: BBP 2. Biological effects at various doses of BBP without exogenous adipogenic stimuli
Al-Abdulla et al., 2022 [158]	MIN-6 cell line (pancreatic cells)	1. ↓ viability of cells after 24 exposure at 1 μΜ 2. ↑ mRNA level of SUR1, GLUT2 at 10 μΜ 3. ↓ GSIS (20 μΜ glucose) 4. ↓ insulin content at 1 μΜ	1. Animal in vitro model 2. Pthalate: DEHP 3. Dose: 100 pM, 1 nM, 10 nM, 100 nM, 1 µM, 10 µM
Schaffert et al., 2022 [141]	SGBSs (pre-adipocytes)	1. DINP: - ↑ binding to PPARγ - (-) PPARγ activation - (-) lipid accumulation - ↑ adipsin (10 µM) - Mature adipocytes: * 10 µM: ↑ MCP-1, LAP3, GPX1 * 10 nM: ↑ GPX8, GSR * 10 nM, 10 µM: ↑ LEP, GPX4 * 10 nM, 10 µM: ↓ adiponectin 2. DPHP: - ↑ binding to PPARγ - (-) PPARγ activation - Undifferentiated cells: * (-) lipid accumulation * ↓ MCP-1 (10 nM, 10 µM) - Mature adipocytes: * ↓ lipid accumulation (10 µM, 25 µM, 50 µM, 100 µM) * 10 µM: ↑ LEP, MCP-1, LAP-3, GPX4, GPX8, adipsin * 10 nM: ↑ GSR * 10 nM, 10 µM: ↑ GPX1, GSTO1 * 10 nM, 10 µM: ↓ adiponectin 3. MHINP: - ↑ binding to PPARγ (100 µM, 200 µM, 400 µM) - ↑ PPARγ activation (1 µM) - Undifferentiated cells: * ↑ pre-adipocyte differentiation, lipid accumulation (10 µM, 25 µM, 50 µM, 100 µM) * 10 µM: ↑ LEP, PLIN1, GPD1, FASN, FABP4, FABP5 * 10 nM: ↓ MCP-1 * 10 nM, 10 µM: ↑ adipsin - Mature adipocytes: * 1 µM: ↑ lipid accumulation * 10 µM: ↑ LAP3, adipsin * 10 nM: ↑ GSR, GPX8 * 10 nM, 10 µM: ↑LEP, MCP-1, GPX1, GPX4, GSTO1 * 10 nM, 10 µM: ↓ adiponectin 4. OH-MPHP: - ↑ binding to PPARγ - ↑ PPARγ activation - Undifferentiated cells: * ↑ pre-adipocyte differentiation, lipid accumulation (10 µM, 25 µM, 50 µM) * ↑ LEP, GPD1, FASN, FABP4, FABP5 (10 µM) - Mature adipocytes: * 10 µM: ↑ LAP3, GPX1, GPX4, GPX8, adipsin * 10 nM, 10 µM: ↑ LEP, GSR, MCP-1, GSTO1 * 10 nM, 10 µM: ↓ adiponectin * 10 nM, 10 µM, 25 µM, 50 µM, 100 µM: ↓ lipid accumulation	1. Human in vitro model 2. Phthalates: DINP, DPHP, MHINP, OH-MPHP 2. Obesogenic effects at - DINP: 10 nM, 10 µM, - DPHP: 10 nM, 10 µM, 25 µM, 50 µM, 100 µM - MHINP: 10 nM, 10 µM, 25 µM, 50 µM, 100 µM, 200 µM, 400 µM - OH-MPHP: 10 nM, 10 µM, 25 µM, 50 µM, 100 µM, 200 µM, 400 µM

Abbreviations: AACS: acetoacetyl-CoA synthetase; AP2: adipocyte protein 2; BPA: bisphenol A; BBP: benzyl butyl phthalate; CCL20: chemokine (C-C motif) ligand 20; C/EBPα: CCAAT/enhancer-binding protein α; CD36: fatty acid translocase; CIDEA: cell-death-inducing DNA fragmentation factor-alpha-like effector A; DCHP: bis(2-propylheptyl) phthalate; DBP: dibutyl phthalate; DEHP: di(2-ethylhexyl) phthalate; DINP: diisononyl phthalate; DPHP: bis(2-propylheptyl) phthalate; ED: endocrine disruptor; ERα: estrogen receptor α; ERβ: estrogen receptor β; ERK1/2: extracellular-signal-regulated protein kinase 1/2; FABP4: fatty-acid-binding protein 4; FABP5: fatty-acid-binding protein 5; FABP4/AP2: fatty-acid-binding protein 4/adipocyte protein 2; FASN: fatty acid synthase; FOXO1: forkhead box protein O1; GLUT2: glucose transporter type 2; GLUT4: glucose transporter type 4; GPD1: glycerol-3-phosphate-dehydrogenase; GPX1: glutathione peroxidase 1; GPX4: glutathione peroxidase 4; GPX8: glutathione peroxidase 8; GSIS: glucose-stimulated insulin secretion; GSR: glutathione-disulfide reductase; GSTO1: glutathione S-transferase omega-1; IFN-γ: interferon-γ; IL: interleukin; IR-β: insulin receptor subunit β; JAK/STAT: Janus kinase/signal transducer and activator of transcription; JNK: c-Jun N-terminal kinase;LAP3: leucine aminopeptidase 3; LEP: leptin; LPL: lipoprotein lipase; LSD-1: lysine-specific demethylase-1; MCP-1: monocyte chemoattractant protein-1; MDA: malondialdehyde; MEHP: mono-2-ethylhexyl phthalate; MHINP: monohydroxy isononyl phthalate; N2A: Neuro-2A cells; NAD: nicotinamide adenine dinucleotide; Nampt: nicotinamide phosphoribosyltransferase; NF-kB: nuclear factor kappa-light-chain-enhancer of activated B cells; OH-MPHP: 6-hydroxy monopropylheptyl phthalate; PDK4: pyruvate dehydrogenase kinase 4; PPARα: peroxisome-proliferator-activated receptor α; PPARγ: peroxisome-proliferator-activated receptor γ; PKB/Akt: protein kinase B/AKT; PLIN1: perilipin-1; ROS: reactive oxygen species; SCOT: succinyl-CoA-3-oxoacid CoA-transferase; SIRT1: sirtuin 1; SIRT3: sirtuin 3; SOD: superoxide dismutase; SUR1: sulfonylurea receptor 1; TBBPA: tetrabromobisphenol A; TGs: triglycerides. ↑ increase, ↓ decrease.

## 7. Evidence from Animal Studies Linking BPA and Phthalates to Obesity

A significant number of in vivo studies in animals propose an association of BPA exposure with adipogenesis (Table 2). In fact, BPA exposure during pregnancy and lactation may provoke weight gain in the postnatal period, as well as an increase in the adipose tissue mass in the offspring. Interestingly, these features occur at low maternal BPA concentrations in comparison to the environmental levels of BPA. According to Wei et al., these unfavorable outcomes may become more evident when animals are exposed to a high-fat diet, and this is thought to be attributed to impaired glucose tolerance [159]. Surprisingly, elevated BPA levels in plasma do not seem to lead to maternal weight gain and increased body fat, due to the augmented secretion of α-fetoprotein during gestation. BPA’s binding to α-fetoprotein may abrogate its protective effects on the developing fetus, which are normally achieved through its binding to estrogen [160]. On the other hand, several in vivo studies have focused upon the potential adverse effects of different phthalates regarding obesity, implicating a wide range of proposed mechanisms. Most of these obesogenic features are investigated using rodents or zebrafish animal models and are thought to be dose-dependent or gender-related, respectively. Notably, effects on body weight and visceral adipose tissue mass have been reported at environmentally relevant doses, suggesting that phthalate exposure may be associated with obesity risk in humans.

Obesogenic features of BPA are correlated with its ability to exhibit hormone-like properties by interfering with specific receptors. In particular, BPA resembles the effects of estrogen, via binding to ERα and Erβ receptors. In such cases, attenuated adiponectin secretion, as well as increased adipocyte differentiation and lipid accumulation, may be seen [109]. In addition, BPA exposure may disable the beneficial impact of some adipokines in lipolysis, resulting in impaired lipid metabolism and difficulty in maintaining normal body mass. Existing in vivo experimental evidence has shown the adipogenic effects of BPA exposure due to its ability to interact with PPAR. BPA modulates PPAR-γ signaling, resulting in augmented expression of genes that induce adiposity [161]. On the other hand, BPA seems to inhibit the expression of PPAR-α, thus promoting liver TG deposition via alterations in lipid oxidation [113]. PPAR-γ activation is suggested to play an essential role in adipogenesis induced by phthalate exposure as well. According to Hao et al., DEHP injection in C57BL/6J mice increased adipose tissue weight due to the enhanced expression of adipogenic transcriptional factors PPAR-γ, C/EBP and SREBF1 [162]. In addition, DEHP exposure in female mice, for a period of 10 weeks, is associated with weight gain and increased adipose tissue mass, whereas insulin sensitivity impairment and maladaptive adipose tissue function were observed. Changes in circulating and tissue adiponectin, as well as the augmented expression of ERs and the activation of PPAR-γ, may be involved [163]. PPAR-γ activation seems to be also involved in adipogenesis, following MEHP exposure, resulting in the differentiation of pre-adipocyte-like adipocytes into lipid-laden and insulin-responsive adipocytes [164]. Interestingly, Zhuang et al. pointed out the role of the transforming growth factor (TGF)-β signaling pathway in adipocyte differentiation, via the stimulation of the adipogenic factors PPAR-γ and CEBPα, in contrast to the estrogen signaling pathway, which had no effect [165].

Recently, Tian et al. reported that the activation of CB1 may be a potential sequela of BPA exposure, resulting in increased appetite and ultimately obesity. The CB1 receptor is expressed in a variety of tissues, including nervous system and fat tissues, and may have an essential role in regulating food intake and energy storage [113]. Furthermore, BPA exerts its harmful effects on the central nervous system through the stimulation of orexigenic hypothalamic neuropeptides, AgRP and NPY [166]. Similar data suggest that DEHP, following 5 weeks of exposure, favors food intake and increased body weight in male C3H/He mice, probably due to the augmented expression of hypothalamic neuropeptides involved in the appetite regulation and the synergistic effects of hypothyroidism and hypothalamic leptin resistance [135]. Evidence from animal studies has underscored that exposure to phthalates may lead to alterations in lipid metabolism, resulting in dysregulation of energy homeostasis (Table 2). In zebrafish, DEHP at low doses alters the expression of liver genes associated with fatty acid metabolism, consequently leading to non-alcoholic fatty liver disease (NAFLD). Notably, these effects were absent following 17α-ethinylestradiol exposure, which typically lacks estrogen features [167]. On the other hand, high concentrations of DEHP may weaken diet-related obesity in mice, potentially via PPAR-α-dependent stimulation of hepatic fatty acid catabolism. Notably, this effect is observed in mice, but not in humans [97]. In rats, DEHP at the dose of 5–200 mg/kg daily alters the lipid profile, resulting in reduced concentrations of high-density lipoproteins (HDLs) and increased levels of total cholesterol and triglycerides [168]. Furthermore, MEHP has been proposed to boost the pathological progression of liver steatosis in a zebrafish animal model, upon co-administration with ethanol. In such cases, DNA damage and apoptosis seem to be mediated via CYP4A and alcohol dehydrogenase (ADH) involvement [169].

Exposure to BPA may also have an impact on the intestinal microbiota due to its ability to alter LPS and gut SCFA levels. Consequently, impaired lipid homeostasis and chronic low-grade activation of the inflammatory cascade may develop [109]. In accordance with this observation, long-term DEHP exposure may provoke gastrointestinal dysbiosis, thus increasing fat storage in zebrafish, as a result of the disrupted expression of genes associated with lipid metabolism [170]. Early-life BPA exposure could account for transgenerational epigenetic alterations, which may have an impact on obesity risk, across several generations. These effects are believed to occur due to specific changes in the DNA methylation pattern of genes modulating metabolic pathways. Susiarjo et al. showed that maternal exposure to BPA in C57BL/6 mice may induce metabolic abnormalities due to the overexpression of the imprinted insulin-like growth factor 2 (IGF-2) gene and increased DNA methylation at the IGF-2 locus [171]. Similar to BPA, phthalates also seem to facilitate epigenetic transgenerational effects on obesity. Prenatal phthalate exposure may modify DNA methylation at loci near genes, resulting in alterations in metabolic hormone signaling pathways. Specifically, Bis(2-ethyhexyl) tetrabromophthalate (TBPH) and its metabolite TBMEHP may induce demethylation of the PPAR-γ promoter DNA, leading to changes in lipid metabolism in early larval stages of zebrafish. As a result, lipid lipolysis and energy homeostasis may be disrupted [172].

In agreement with in vitro experimental data, potential in vivo obesogenic effects of BPA and phthalates seem to be mediated by mechanisms involving the activation of adipogenic transcriptional factors, stimulation of specific receptors and enhancement of the inflammatory cascade. In addition, disruption of gut dysbiosis, as well as transcriptional epigenetic alterations associated with DNA methylation modification, may also be involved.

**Table 2:** Major animal studies showing associations between BPA and phthalates and obesity.

Author, Year	Type of Animal Used	Main Findings	Remarks
Bisphenol A and obesity			
Pu et al., 2017 [161]	Primiparous female sheep	1. ↑ differentiation rate in adipocytes 2. ↑ mRNA expression of PPARγ in fetal adipose tissue 3. ↑ expression of FABP4, GLUT4 and SOX6 in the offspring 4. ↑ gene expression of GR, ESR1, ESR2 and ERRα	1. Type of exposure: sc 2. Exposure duration: 147 days 3. Daily BPA dose: 0.5 mg/kg
Desai et al., 2018 [160]	12-week-old female Sprague–Dawley rats	1. ↑ body weight 2. ↑ mass of adipose tissue 3. Hypertophic adipocytes in male offspring 4. ↑ expression of PPARγ 5. ↑ TNF-α and CD68 in adipose tissue	1. Findings occurred in the offspring 2. Type of exposure: drinking water 3. Exposure duration: 2 weeks before mating up to weaning 4. Daily BPA dose: 5 mg/L
Stoker et al., 2019 [173]	90-day-old female Wistar rats	1. ↑ food intake 2. ↑ epididymal and perirenal fat deposition 3. ↑ fasting serum glucose and leptin in male mice 4. ↑ expression of hypothalamic orexigenic neuropeptides in male mice	1. Findings occurred in the offspring 2. Type of exposure: drinking water 3. Exposure duration: pregnancy day 9 to weaning 4. Daily BPA dose: 50 µg/kg
Lin et al., 2019 [174]	3-week-old male Wistar rats	1. ↑ fat deposition (visceral, liver) 2. ↑ TCHOL, LDL, TGs 3. ↓ HDL 4. ↑ TNF-α, IL-17 5. ↑ mRNA of SREBP1 and ACC1 6. ↑ TLR4 and NF-κB in the liver 7. ↓ HSL, ERα and ZAG in the liver	1. Type of exposure: drinking water 2. Exposure duration: 8 weeks 3. Daily BPA dose: 1 µg/mL
Tian et al., 2021 [113]	5-month-old wild-type adult male Danio rerio	1. ↑ weight gain, length, food intake 2. ↑ lipid accumulation in liver 3. Microvesicular fatty changes, hepatocyte ballooning, infiltration with inflammatory cells 4. ↑ of CB1 5. ↑ of insulin signaling pathways 6. ↓ expression of PPARα in adipose tissue and liver 7. ↓ gpr55	1. Type of exposure: water in static system 2. Exposure duration: 28 days 3. Daily BPA dose: 20, 100 and 500 μg/L
Shih et al., 2021 [175]	15-week-old female Sprague–Dawley rats	1. ↑ abdominal lipid weight up to 77% in female offspring 2. ↑ TCHOL, LDL, TGs 3. ↓ HDL 4. ↑ leptin 5. ↑ of *Prevotella, C. perfringens, C. ruminantius* in feces	1. Type of exposure: oral gavage 2. Exposure duration: 6th-36th day after pregnancy 3. Daily BPA dose: 50 µg/kg
Zhuang et al., 2023 [165]	7-week-old male and female ICR mice	1. ↑ weight gain in the offspring 2. ↑ size of adipocytes 3. ↓ insulin sensitivity 4. No obesogenic effects via estrogen signaling pathway 5. Obesogenic effects via TGF-β signaling pathway	1. Type of exposure: drinking water 2. Exposure duration: 7 days treatment up to delivery 3. Daily BPA dose: 0.5 μg/kg
Phthalates and obesity			
Hao et al., 2013 [162]	C57BL/6J mice	1. ↑ expression of PPARγ, aP2, LPL and FAS 2. ↑ expression of C/EBP, Srebf1 3. ↑ glucose, TCHOL, TGs in serum 4. Obese phenotype only at the dose of 0.25 mg/kg in female offspring 5. ↑ weight gain in male offspring	1. Type of phthalate: DEHP 2. Ip DEHP at the dose of 0.5 mg/kg in six-week-old male mice 3. Female mice: - Type of exposure: gavage - Exposure duration: from day 12 of gestation until day 7 of lactation - Daily DEHP dose: 0.05, 0.25 or 0.5 mg/kg
Klöting et al., 2015 [163]	Obesity-resistant 129S6 mice	In female (but not in male) mice: 1. ↑ weight gain 2. ↑ fat mass 3. ↓ insulin tolerance 4. ↓ Pparg and adiponectin in scAT 5. ↑↑ Esr1 protein levels in SC and visceral adipose tissue 6. (-) TCHOL, TGs in serum 7. ↑ in phospholipid and carnitine	1. Type of phthalate: DEHP 2. Type of exposure: oral 3. Exposure duration: 10 weeks 4. Daily DEHP dose: 0.05 mg/kg
Lv et al., 2016 [135]	Male C3H/He mice	1. ↑ food intake, adipogenesis and weight gain in all exposure groups except for 0.05 mg/kg 2. Interruption in hypothalamic appetite-related neuropeptides: - ↑ expression of AgRP in all groups - ↑ expression of NPY at 50 and 200 mg/kg - ↓ expression of POMC at 200 mg/kg 3. Hypothalamic leptin resistance resulting in hypothyroidism 4. ↓ WAT lipid metabolism at 0.5 mg/kg 5. ↑ WAT lipid metabolism at 50 and 200 mg/kg	1. Type of phthalate: DEHP 2. Type of exposure: gavage 3. Exposure duration: 5 weeks 4. Daily DEHP dose: 0.05, 0.5, 5, 50 and 200 mg/kg
Zhang et al., 2020 [176]	C57BL/6 J male and female mice	1. Weight gain in male mice on HFD at 3 mg/kg/d 2. At the dose of 3 mg/kg/d: - ↑ activation of SREBP1 - (-) of SREBP2, PPARγ - ↑ expression of downstream regulatory genes of SREBP1 (FAS, ACC, HMGCR) 3. ↓ insulin tolerance in male mice between HFD + BBP3 and HFD groups	1. Type of phthalate: BBP 2. Type of exposure: oral 3. Exposure duration: 16 weeks 4. Daily BBP dose: 4 μg/kg, 169 μg/kg, 3 mg/kg, 50 mg/kg
Guo et al., 2020 [172]	Zebrafish embryos 0.75 hpf	- ↑ expression of PPARγ due to the following: 1. Significant regional DNA demethylation 2. Upregulation of tet1 and tet2 gene transcription) - As a result, ↓ TCHOL and TGs due to ↑ expression of downstream genes involved in lipid metabolism	1. Type of phthalate: TBPH and TBMEHP 2. Type of exposure: glass Petri dish containing 100 mL of TBPH or TBMEHP 3. Exposure duration: until 72 hpf 4. Daily TBPH and TBMEHP dose: 0.2–2000 nM
Buerger et al., 2020 [177]	Zebrafish (Danio rerio)	GI dysbiosis in the OF + DEHP group as a result of the following: - ↑ of Bacteroidetes - ↑ of UFAs - ↑ lipid metabolism - ↓ carbohydrate metabolism - ↓ glycerolipid metabolism - ↓ glycerophospholipid metabolism - ↓ carbohydrate, galactose, inositol phosphate, taurine and hypotaurine metabolism	1. Type of phthalate: DEHP 2. Type of exposure: oral 3. Exposure duration: 60 days 4. Daily DEHP dose: 3 mg/kg

Abbreviations: ACC1: acetyl-CoA carboxylase 1; AgRP: Agouti-related protein; AP2: adipocyte protein 2; BPA: bisphenol A; CB1: endocannabinoid receptor type 1; CD68: cluster of differentiation 68; C/EBP: CCAAT/enhancer-binding protein; ESR1: estrogen receptor 1; ESR2: estrogen receptor 1; ERRα: estrogen-related receptor α; FABP4: fatty-acid-binding protein 4; FAS: fatty acid synthase; GLUT4: glucose transporter type 4; GR: glucocorticoid receptor; HDL: high-density lipoprotein cholesterol; HSL: hormone-sensitive lipase; IL-17: interleukin-17; LDL: low-density lipoprotein cholesterol; LPL: lipoprotein lipase; NPY: neuropeptide Y; NF-κB: nuclear factor kappa-light-chain-enhancer of activated B cells; PPARγ: peroxisome-proliferator-activated receptor γ; POMC: proopiomelanocortin; SC: subcutaneous; SOX6: SRY-box transcription factor 6; SREBP1: sterol regulatory element-binding transcription factor 1; TNF-α: tumor necrosis factor-α; TCHOL: total cholesterol; TGF-β: transforming growth factor-β; TGs: triglycerides; TLR4: toll-like receptor 4; WAT: white adipose tissue; ZAG: ZAG adiponectin. ↑ increase, ↓ decrease.

## 8. Evidence from Human Studies Linking BPA and Phthalates to Obesity

There is an abundance of epidemiological studies assessing the effects of BPA and phthalates on human adiposity (Table 3). Urinary and, to a lesser extent, serum concentrations of BPA and different phthalate metabolites have been used as surrogates of exposure. In several studies, urinary measurements have been adjusted for creatinine concentration to account for the confounding effect of urine dilution due to the individual hydration state.

### 8.1. Bisphenol A and Obesity

In general, the majority of human cross-sectional epidemiological studies in adult patients demonstrate a direct association between BPA measurements in urine and generalized (indexed by the BMI) as well as abdominal (based on WC measurements) obesity [182,189,191,194,196,197]. This observation is further strengthened by the findings of longitudinal studies [190,192] demonstrating accelerated weight gain and higher incidence of central obesity among individuals with higher urinary or serum BPA concentrations [181]. The findings regarding this relationship are not equivocal, since other studies with similar methodologies have failed to demonstrate any substantial associations [183,187]; however, existing meta-analyses of the available clinical studies point towards a consistent association of higher BPA measurements with the prevalence of overweight and generalized or central obesity [178,185,186], while one study has demonstrated the presence of an inverse U-shaped dose–effect relationship between serum BPA and incident overweight/obesity in individuals with normal weight [181]. Similar associations have also been found between BPA and childhood/adolescent obesity [179,184,187,188,193,195] and are additionally supported by relevant meta-analyses [215] despite the presence of studies with neutral results [183]. Importantly, even though some disparity among studies exists in this regard, the overall association of BPA exposure with obesity risk likely affects both genders to a significant degree. The results of a recent umbrella meta-analysis have strengthened this notion; however, a considerable heterogeneity among studies in males was noted, rendering the association between BPA and obesity more convincing for females [178]. Furthermore, the available observations have been conducted in ethnically diverse populations, allowing for the generalizability of the findings. Few observational studies have pointed towards race-dependent effects of BPA on obesity risk [193,195], while in others, the risk appears to be consistent across different ethnic backgrounds [194]. The studies on the impact of BPA exposure during gestation on infantile birth weight have yielded inconclusive results, demonstrating either neutral effects, restricted fetal growth or abnormally high birth weight [216,217], with most data favoring a relationship with growth restriction, especially when exposure during early pregnancy is taken into account [218]. These findings do not contradict the bulk of evidence favoring an obesogenic effect of BPA, but rather demonstrate the overall deleterious endocrine-disrupting properties of the compound.

Apart from measures of adiposity, exposure to BPA has been associated with obesity-related metabolic perturbations and a more adverse overall metabolic profile, including insulin resistance, hyperglycemia and overt T2DM [219,220,221] independently of traditional risk factors, arterial hypertension [222] and dyslipidemia [223,224]. It is unclear whether these observations emerge as direct consequences of BPA exposure or are indirectly mediated by its putative obesogenic effects.

Due to the overall adverse safety profile of BPA, the use of other bisphenols such as BPS, BPF or BPAF in the manufacture of plastics has been increasing. However, these compounds are not free of endocrine-disrupting properties [225]. With respect to obesity, there are increasing reports of an association with BPF, PBS or PBAF exposure [180,182,187,188,189], occasionally more robust than that of BPA [180,187], although other studies have not ascertained significant effects of BPF or BPS exposure on obesity risk [183].

### 8.2. Phthalates and Obesity

Most available cross-sectional studies have demonstrated a direct association between one or more of the numerous measured phthalate metabolites and measures of adiposity [78,203,204,208,212,214]. Supportive evidence has also been provided by prospective observational studies, which have generally demonstrated associations between phthalate urine [192,202,205,209] or serum [213] concentrations and prospective weight or fat mass over follow-up durations as prolonged as approximately 10 years. Another prospective study ascertained a significant association of baseline urine phthalates with weight gain in the short (after 3 years) but not long term (6 years) [208], suggesting that the effect of phthalates on weight gain may be short-lived and may vary with changing levels of exposure over time. Interestingly, increased baseline phthalate concentrations were also associated with impaired weight loss during a dietary intervention, suggesting obesogenic properties [226]. It should be noted that the sum of available prospective studies has based their associations on momentary urinary measurements at baseline, which arguably do not necessarily reflect the cumulative exposure over time.

Disparities also remain as to whether the effects of specific phthalate compounds are sex- or age-group-dependent [210,213,214], while evidence also shows that higher adiposity at the time of phthalate measurement facilitates the obesogenic effects of phthalates [201]; it is unclear, however, whether the latter is an actual priming effect of already-existing obesity or if it could merely reflect a confounding effect of higher cumulative phthalate exposure and obesogenic effect also in the time period preceding the measurement. Available meta-analyses indicate that the presence of an association between phthalate exposure and obesity is likely, although the reported results are highly heterogeneous. Furthermore, the presence of sex-specific effects or publication bias in available reports cannot be excluded [200,207].

Certain reports have also demonstrated similar associations in adolescents [214] while the evidence for an association with childhood obesity are conflicting [204,214]—at least regarding the presence of effects independent of other confounding factors pertinent to diet and/or physical activity [182]. Similarly, conflicting results appear in prospective studies investigating the effects of intrauterine exposure to phthalates (based on concentrations in maternal urine during pregnancy) and infantile obesity, with some demonstrating an adverse relationship between maternal exposure and offspring weight gain [206], some demonstrating neutral [210] or even beneficial effects [210,211] and others demonstrating phthalate-compound-specific effects [209]. A recent meta-analysis indicated an overall association of prenatal exposure to the most broadly used di-(2-ethylhexyl)-phthalate with decreased BMI z-scores in infants but no impact on fat mass, suggesting that the correlation between phthalate exposure and BMI is mediated by decreased muscle growth [227].

## 9. Perspectives, Controversies and Challenges

Even though the bulk of evidence tends to suggest overall adverse effects of exposure to BPA and phthalates on obesity-related endpoints, the methodological limitations of available observations should also be considered. The majority of available clinical studies are of cross-sectional nature, hence not allowing for concrete conclusion, although existing prospective epidemiological analyses have generally yielded compatible results. Furthermore, practically, the majority of studies have based their results on single momentary measurements of these pollutants, particularly in urine. The use of single measurements for nonpersistent chemicals is poor [228]. In observational studies, multiple repeated specimens need to be collected for EDC determination over an extended chronic period of toxicological relevance, i.e., before and during at-risk temporal windows for a disease, and with consideration of exposure patterns [80,228]. The probability of background contamination of samples during handling, conservation or even laboratory measurement constitutes another source of potential bias [229,230,231]. However, despite these theoretical considerations, it appears that, at least in the case of BPA assays, contamination is in fact negligible in most laboratories [232], and in any case, this does not itself present a sufficient ground for discrediting the ascertained associations between BPA or phthalate measurements and adverse health outcomes, including obesity. Another factor that should be considered when examining the effects of exposure on adiposity and other metabolic readouts is that BPA and phthalate exposure also occurs during the consumption of fat- and sugar-rich, highly caloric, palatable meals of low nutritional value (“junk food”) [233]; hence, the epidemiological associations could potentially be mere reflections of an unhealthy dietary lifestyle. Certain studies have taken dietary factors into account as potential confounders [179,192,199]; nevertheless, this remains a considerable potential source of bias that should not be overlooked in epidemiological studies. Lastly, exposure to multiple chemicals can occur from single sources; therefore, a causal nature of the observed correlations cannot be readily assumed, while the estimation of combined effects of multiple exposures becomes particularly challenging for studies of observational nature. 

In any case, and even though available data from human studies are not unanimous for an unequivocal obesogenic effect of BPA or phthalate exposure, the majority of available evidence, coupled with the results of preclinical and in vitro studies, collectively present a strong argument in support of this notion. Furthermore, the fact that both compound groups have been associated with an adverse profile of other relevant cardiometabolic risk determinants such as insulin resistance and T2DM [234,235,236,237,238], dyslipidemia [223,239,240] and hypertension [241,242] renders the exposure to these ubiquitous chemicals a reasonable source of public health concern.

A gradual replacement of both BPA and phthalates by alternative compounds in the production of plastic has been undertaken on a large scale in order to diminish the proven and putative adverse health effects of exposure. For example, Canada has banned the marketing of baby bottles containing BPA since 2010 [243]. This has led to the increasing use of bisphenols other than BPA, which are, however, not free of endocrine-disrupting pharmacological actions [225]. On the contrary, obesogenic properties [187,188,189] and possible links to an increased cardiovascular risk [244,245,246] have also been attributed to BPF and BPS. Collectively, all these issues mandate the displacement of the sum of bisphenol compounds and their replacement by alternative plasticizers, such as those derived from plant biomass [243]. Accordingly, viable alternatives to phthalates include adipates, diisononyl cyclohexane-1,2 dicarboxylate (DINCH) and bis-2-ethylhexyl terephthalate, which exhibit a less hazardous profile, although human exposure has been steadily increasing in the latest years [247,248].

Currently, the replacement of phthalates and bisphenols constitutes a field of active applied research. Due to the ubiquitous use of plastics and the subsequent exposure hazard to humans, there is a pressing need for meticulous testing of candidate compounds in preclinical and epidemiological studies in order to establish their safety with regard not only to potential endocrine disruption but also to overall long-term health effects.

## 10. Conclusions

Based on the available data from mechanistic, animal and epidemiological human studies including meta-analyses, the weight of evidence points towards the contribution of EDCs to the etiopathogenesis of obesity. Moreover, these ubiquitous chemicals such as BPA and phthalates as well as their analogs have been linked to obesity-associated disorders, namely insulin resistance, T2DM, dyslipidemia, hypertension and, similar to advanced glycation end products (AGEs), female hormone imbalances [249,250]. Undoubtedly, the type of diet, caloric intake and the lack of somatic exercise are crucial factors in the obesity pandemic; however, the decrease in exposure to obesogenic EDCs, particularly during susceptible time windows such as prenatal, neonatal, pubertal and reproductive periods, may also contribute to decreasing excess body weight and its associated implications in the population. It is important to underscore that similar to hormones, EDCs may act in several organs at very low levels promoting time- and tissue-specific effects, including metabolic, endocrine, neurological, reproductive and transgenerational implications. Besides known EDCs, there are many chemical compounds or complex mixtures presenting hormonal/growth factor signaling (metabolism, carcinogenesis)-disrupting actions, such as (anti)estrogenic or antiandrogenic actions and deregulation of the IGF-1–IGF-1 receptor axis [251,252], particularly in plastic bottled water [253,254]. It is of paramount importance that all chemical compounds should undergo testing for their endocrine-disrupting properties at low levels by using in silico, mechanistic and animal studies before marketing. International standardization is required for the biomonitoring and evaluation of EDCs to improve policymaking and understanding regarding the actual exposure of people to EDCs and their potential implications for health. Limitations of their use or substitution as well as educational programs in maternity clinics about the use of EDCs and their obesogenic and metabolic consequences represent important preventive measures. An example of substitution of EDCs in water bottles could be the use of alternative materials such as glass, aluminum or stainless steel. Although difficult to use, glass is a safe reusable bottle material for storing both food and liquids because it is chemical-free, constructed from natural products and dishwasher-safe. Before any regulatory action, subjects should be informed so that they may decrease or avoid exposure to EDCs as much as possible for themselves and their children.

## Figures and Tables

**Table 3:** Major studies depicting associations between endocrine disruptors and obesity.

Author, Year	Study Design/Population	Main Findings	Comments
Bisphenol A and obesity			
Lin et al. (2023) [178]	Umbrella review of systematic reviews with meta-analyses on the association of BPA exposure with multiple outcomes, including obesity	- Higher BPA exposure significantly associated with obesity risk in both sexes (females: OR 1.51; males: OR 1.88) - Significant associations with generalized and abdominal obesity (OR 1.22 and 1.41, respectively) as well as overweight in adults (OR 1.25) - Significantly increased risk for type 2 DM (OR 1.28)	Higher BPA exposure associated with obesity in children and adults, with less heterogeneity among studies in females
Deodati et al. (2023) [179]	Case–control study among n = 122 children (n = 66 and n = 56 with and without obesity, respectively) matched for age and gender	- Significantly higher creatinine-adjusted urinary BPA concentrations in obesity than normal weight (10.77 vs. 5.50 µg/g, respectively) among girls, but not boys. - Significantly higher risk of obesity in children with BPA levels above the median eating packaged food (OR = 11.09)	Potential gender-specific relationship between BPA exposure and higher odds of childhood obesity in girls
Chen M et al. (2023) [180]	Cross-sectional study among n = 426 children aged 7 years old	- Urinary concentrations of BPA substitutes BPS, BPAF exhibit a significant positive association with BMI, WC, overweight/obesity only among boys - No associations between adiposity measures and BPA or other substitute compounds	Associations between BPS, BPAF, but not BPA exposure and obesity in boys
Bi J et al. (2022) [181]	Prospective observational study including n = 796 individuals with normal weight, among whom 133 developed overweight or obesity during follow-up	- Presence of a statistically significant inverted U-shaped relationship between serum BPA and incident overweight/obesity - Significant positive correlation between log10-BPA and increase in waist-to-hip ratio - Serum adiponectin mediates 46% of association between BPA and incident overweight/obesity	Non-monotonic relationship between baseline BPA and incident overweight/obesity among individuals with normal weight, potentially indirectly mediated by adiponectin
Choi et al. (2022) [182]	Cross-sectional study including 1046 adult participants in NHANES (2013–2016) and 3268 adult participants of the Korean National Environmental Health Survey (2015–2017)	Those in the higher urinary BPA tertiles had significantly higher odds for obesity (OR = 1.58 and 1.41 for 3rd and 2nd vs. 1st tertile, respectively) Similar associations for urinary BPF and BPS	Exposure not only to BPA but also to substitutes BPF and BPS is associated with adult obesity
Gajjar et al. (2021) [183]	Prospective observational cohort of n = 212 children with urinary BPA and BPS measurements at 8 years and body composition assessments at 8 years (bioimpedance) and 12 years (DXA)	No evidence for a synchronous or prospective association of urinary BPA or BPS with increased adiposity	
Wu et al. (2020) [184]	Cross-sectional study including n = 2372 children and adolescents (aged 6–19) participating in NHANES	BPA levels significantly associated with higher weight in a statistical approach implementing weighted quantile sum statistical model but not in other approaches	Evidence for an association between BPA exposure and childhood/adolescent obesity
Ribeiro et al. (2020) [185]	Meta-analysis of studies investigating BPA exposure and multiple adverse health outcomes, including obesity	- BPA is significantly associated with overweight (OR 1.254), obesity (OR 1.503) and increased WC (OR 1.503) in adults - OR 1.8 for childhood obesity	Positive association between BPA exposure and generalized as well as abdominal obesity
Wu et al. (2020) [186]	Meta-analysis of 10 observational studies	- Statistically significant dose–response positive relationship between BPA and overweight/obesity risk in both sexes - 11% increase in obesity risk for every 1 ng/mL of BPA	Continuous positive relationship between BPA and obesity irrespective of sex
Jacobson et al. (2019) [187]	Cross-sectional study including n = 1831 children and adolescents (aged 6–19) participating in NHANES	Urinary BPS and BPF but not total bisphenols or BPA were significantly associated with, particularly abdominal obesity	Substitute bisphenol exposure may predispose individuals to childhood/adolescent obesity
Liu et al. (2019) [188]	Cross-sectional study including n = 745 children and adolescents (aged 6–17 years) participating in NHANES	Urinary BPA (OR 1.74) and BPF (OR 1.54) are significantly associated with obesity, with stronger associations between boys Similar findings for abdominal obesity	Urinary BPA and its substitute BPF associated with obesity, particularly in boys
Zhang et al. (2019) [189]	Cross-sectional study including n = 1269 adults participating in NHANES	Among other chemicals, increased urinary BPA and BPS are significantly associated with higher obesity prevalence	Exposure to BPA, and BPS may predispose individuals to adulthood obesity, although the authors recommend considering the joint effects of different chemical exposures
Hao et al. (2018) [190]	Prospective study (mean follow-up: 4 years) among 888 Chinese adults without abdominal obesity at baseline	OR = 2.30 for incident abdominal obesity each unit increase in log [BPA] urinary concentration after adjustment for confounding factors Individuals in the lowest tertile of BPA concentrations had the lowest risk for incident central obesity (ORs 1.73 and 1.81 for those in the 2nd and 3rd tertiles, respectively)	Prospective association of BPA exposure with incident central obesity in Chinese adults
Do et al. (2017) [191]	Cross-sectional analysis of data from n = 4733 adults aged (18 to 79 years)	For each natural-log unit increase in urinary BPA concentration, significant increase of 0.33 kg/m^2^ in BMI and 1.00 cm in waist circumference	Dose–response relationship between BPA exposure and generalized as well as abdominal obesity
Song et al. (2014) [192]	Prospective (10 years) cohort study of 977 women with baseline measurements of urinary BPA and 9 phthalate biomarkers	After adjustment for dietary and lifestyle variables, those in the highest BPA quartile gained on average an additional 0.23 kg/year (0.07–0.38) of body weight during follow-up	BPA exposure is associated with greater longitudinal weight gain in women
Bhandari et al. (2013) [193]	Cross-sectional analysis of data from n = 2200 children and adolescents (aged 6 to 18 years) from NHANES (2003–2008)	- OR for obesity = 2.55 for children in the highest vs. lowest quartile of urinary BPA - Associations more robust among males and non-Hispanic Whites	BPA is associated with childhood/adolescent obesity, with potential gender- and race-specific effects
Shankar et al. (2012) [194]	Cross-sectional analysis of data from n = 3967 adult participants in NHANES (2003–2008)	ORs = 1.69 and 1.59 for generalized and abdominal obesity for the 4th vs. 1st quartile of urinary BPA concentrations, persistent after adjustment for several confounders and consistent among gender and race–ethnic groups	BPA exposure is associated with central and abdominal obesity in both genders and all race groups, irrespectively of traditional risk factors
Trasande et al. (2012) [195]	Cross-sectional analysis of data from n = of 2838 children and adolescents (aged 6–19 years) participating in NHANES	- Lowest prevalence of obesity in the 1st vs. 2nd-4th quartiles of ascending urinary BPA concentrations (10.3% vs. 20.1%, 19.0% and 22.3%, respectively) - Association of BPA and obesity significant in Whites but not Blacks or Hispanics	Association between BPA exposure and obesity likely exhibits race-specific effects
Wang et al. (2012) [196]	Cross-sectional study of n = 3390, aged >40 years	- Those in the highest quartile of urinary BPA concentrations showed significantly higher prevalence of generalized (OR = 1.50) and abdominal obesity (OR = 1.28) - Among participants without overweight or obesity, higher BPA was significantly associated with IR (OR 1.94)	Evidence for a positive association between BPA exposure and obesity, as well as IR among lean individuals
Carwile et al. (2011) [197]	Cross-sectional study including n = 2747 adults (aged 18–74) participating in NHANES (2003–2006)	Higher risk of general (OR 1.74) and abdominal (OR 1.58) obesity among individuals in the highest vs. lowest quartile of urinary BPA concentration	BPA exposure is associated with general and abdominal obesity in US adults
Phthalates and obesity			
Deodati et al. (2023) [179]	Case–control study among n = 122 children (n = 66 and n = 56 with and without obesity, respectively) matched for age and gender	- Early downstream metabolites of Di(2-ethylhexyl) phthalate in urine significantly higher in girls with obesity than normal weight - Significant positive correlation of Di(2-ethylhexyl) phthalate metabolites with serum leptin levels	Significant correlation of certain phthalate metabolites with increased adiposity in girls
Stevens DR et al. (2023) [198]	Prospective study among n = 438 infants from the Healthy Start prospective pregnancy cohort.	- Significant inverse association between maternal urinary mono-benzyl and di- n-butyl phthalate at 28th gestational week and percentage fat mass at birth in male infants	Maternal phthalate exposure in pregnancy is inversely associated with fat mass in male, but not female, infants at birth
Li et al. (2023) [199]	Case–control study among n = 240 children with overweight/obesity (OBE) and n = 240 age- and gender-matched controls	Among 9 phthalates, monomethyl phthalate and monobutyl phthalate were significantly higher in controls than children with overweight/obesity but not after adjustment for physical activity and caloric intake.	No significant differences in phthalate concentrations between OBE and controls
Wu et al. (2022) [200]	Meta-analysis of observational studies for the association between phthalate compounds and obesity in adult and pediatric populations	- Mono-n-butyl-, monobutyl-, monoisobutyl-, monoethyl- and mono(2-ethyl-5-carboxypentyl) phthalate significantly associated with obesity, specific compounds more strongly correlate with general or abdominal obesity - Stronger associations in women and in studies from the United States and Europe	Compound-specific effects on general and abdominal obesity, with potential gender- and study-site-specific effects
Boyer et al. (2023) [201]	Measurement of the concentrations of 9 phthalates in n = 379 pregnant women, in relation to gestational weight gain (difference between pre-pregnancy and median 35.1 weeks weight)	Significant direct association between mono-(3-carboxypropyl) phthalate and mono-n-butyl phthalate was positively associated with gestational weight gain (1.81 kg and 0.77 kg at 35 weeks) interquartile range increase among women with obesity	Phthalate exposure is associated with greater weight gain in pregnancy, particularly among women with obesity at baseline
Vieyra et al. (2023) [202]	Prospective observational study n = 1125 participants of the Woman Health Initiative (WHI) with available urine phthalate measurements and DXA-based estimations of VAT and SAT	Significant positive associations of baseline di-isobutyl phthalate biomarkers, monocarboxy-isononly phthalate, and di(2-ethylhexyl) phthalate with VAT three years later, which persisted after adjustment for SAT	Higher levels of certain urinary phthalate compounds are longitudinally associated with higher VAT over time in postmenopausal women
Milankov et al. (2023) [203]	Cross-sectional study among n = 60 women with PCOS	Total urinary phthalate concentrations significantly positively correlate with BMI, waist circumference, waist-to-height ratio, VAI, FPG and HOMA-R	Increased phthalate exposure associated with obesity, insulin resistance and hyperglycemia in women with PCOS
Wang et al. (2023) [204]	Cross-sectional study among n = 798 students (7–10 years)	Significantly increased risk of abdominal obesity for the fourth vs. first quartile (OR = 5.29 and 3.73) and 273% (OR = 3.73; 95% CI: 1.57, 8.86) of urinary concentrations of monoethyl phthalate and monoisobutyl phthalate	Monoethyl-phthalate and monoisobutyl-phthalate exposure are associated with abdominal obesity in children
Peng et al. (2023) [205]	Prospective observational analysis of n = 1369 women in the Study of Women’s Health Across the Nation Multi-Pollutant Study	Significantly higher levels of spot urinary phthalates (except mono-carboxy-isononyl phthalate) were associated with faster increases in body fat percentage and fat mass, but not total body weight change over time	Urinary phthalate concentrations positively correlated with fat gain in middle-aged women
Kupsko et al. (2022) [206]	Prospective observational study of 514 mother–child pairs in pregnancy until twelve years post-term	Higher maternal urine di (2-ethylhexyl) phthalate metabolites significantly associated with greater odds of high and increasing weight in infants Higher di-isononyl phthalate metabolites significantly associated with greater odds increasing weight in infants	Exposure to certain phthalates during pregnancy exerts a significant impact in infant weight trajectories during childhood
Ribeiro et al. (2019) [207]	Meta-analysis of 29 studies for the association between phthalate compounds and obesity in adult and pediatric populations	- The low number of studies for many phthalate compounds precludes meta-analysis - Statistically significant association solely between mono(2-ethyl-5-carboxypentyl) phthalate and obesity in adults (OR = 1.67)	Positive association between many phthalate compounds and adiposity measures, most formally non-significant; possible publication-bias-related effects
Díaz Santana et al. (2019) [208]	Cross-sectional (n = 997) and prospective (n = 660) observational study among participants of the Woman Health Initiative (WHI)	- Significant positive associations between urinary phthalate biomarker concentrations and obesity in cross-sectional analysis - Baseline urinary mono-(2-ethyl-5-oxohexyl)-, monoethyl-, mono-hydroxybutyl- and mono-hydroxyisobutyl phthalate significantly correlate with weight gain after 3 years - No associations with weight changes at 6 years	Exposure to certain phthalates may predispose individuals to obesity and short-term weight gain
Rodriguez-Carmona, et al. (2019) [209]	Prospective cohort study among n = 178 pregnant women	Higher urinary mono-3-carboxypropyl pthalate is significantly associated with moderately increased weight gain over the next 5.2–10.7 years Higher mono-benzyl phthalate significantly associated with lower weight gain in the same timeframe	Prospective association between phthalate exposure in pregnancy and prospective weight changes in women
Buckley et al. (2016) [210]	Prospective cohort study assessing the fat mass of n = 180 children (4–9 years) in relation to maternal third-trimester urinary phthalate concentrations in pregnancy	- No continuous associations between maternal urinary phthalate concentrations and fat mass in offspring, without apparent gender-specific effects - 3.06% lower fat mass in children in the highest vs. lowest quartile of summed di(2-ethylhexyl) phthalate metabolites	No evidence for an impact of maternal phthalate exposure and increased infantile fat mass
Valvi et al. (2015) [211]	Prospective cohort study of n = 391 mothers with creatinine-adjusted measurements of urinary phthalates in the 1st and 3rd trimesters of pregnancy	High-molecular-weight phthalate metabolites in maternal urine significantly associated with lower BMI z-scores in boys and higher in girls 4–7 years of age	Potential gender-specific effects of maternal phthalate exposure and infantile BMI trajectories
Yaghjyan et al. (2015) [212]	Cross-sectional analysis including n = 6005 women without diabetes participating in NHANES (1999–2004)	- Significant positive associations between monobutylphthalate, mono-2-ethylhexyl- to mono(2-ethyl-5-hydroxyhexyl) phthalate ratio and BMI, WC	Among the observed associations, the higher mono-2-ethylhexyl- to mono(2-ethyl-5-hydroxyhexyl) phthalate ratio may be reflective of slower oxidative metabolism of mono-2-ethylhexyl-pthalate
Song et al. (2014) [192]	Prospective cohort study of n = 977 women with baseline measurements of urinary BPA and 9 phthalate biomarkers	After adjustment for dietary and lifestyle variables, significant albeit moderate positive dose–response relationship between phthalic acid, monobenzyl- and monobutyl-phthalate and weight gain over 10 years	Exposure to certain phthalates is associated with accelerated weight gain in women
Lind et al. (2012) [213]	Prospective cohort study among n = 1016 individuals of 70 years of age	Baseline serum concentrations of mono-isobutyl phthalate and mono-methyl phthalate significantly positively associated with DXA- and abdominal MRI-derived indices of adiposity in women two years later, but not in men	Circulating concentrations of certain phthalates are associated with increased adiposity only in women, suggesting possible sex-specific associations of phthalates with obesity
Hatch et al. (2008) [214]	Cross-sectional analysis of data of n = 4369 NHANES participants (1999–2002)	- Positive trends of BMI and WC across quartiles of concentrations of mono-benzyl, mono-2-ethyl-5-oxohexyl, mono-ethyl, mono-n-butyl, mono-2-ethyl-5-hydroxyhexyl, particularly among males 20–59 years old - Similar trends across mono-ethyl phthalate quartiles in adolescent girls, less strong in adult women - Inverse trend for mono-2-ethylhexyl phthalate in adolescent girls and several inverse associations in adults 60–80 years - No associations in children	Exposure to several phthalates may be associated with increased adiposity, but age-group- and gender-specific effects likely exist
Stahlhut et al. (2007) [78]	Cross-sectional analysis of data of n = 1443 men participating in NHANES (1999–2002)	- Urinary concentrations of monobenzylphthalate, mono(2-ethyl-5-hydroxyhexyl) phthalate, mono(2-ethyl-5-oxohexyl) phthalate and monoethylphthalate are significantly associated with increased waist circumference, after adjustment for confounders - Monobutylphthalate, monobenzylphthalate and monoethylphthalate concentrations exhibit significant positive correlations with HOMA-R	Exposure to certain phthalates is associated with higher abdominal obesity and insulin resistance

Abbreviations: BMI: body mass index; BPA/BPF/BPS: bisphenol A, F and S; DXA: dual X-ray absorptiometry; FPG: fasting plasma glucose; HOMA-R: Homeostatic Model Assessment for Insulin Resistance; NHANES: National Health and Nutrition Examination Survey; PCOS: polycystic ovary syndrome; SAT: subcutaneous adipose tissue; VAI: visceral adiposity index; VAT: visceral adipose tissue, WC: waist circumference.

## Data Availability

Data are contained within the article.

## Abbreviations

ADH: alcohol dehydrogenase; AGEs: advanced glycation end products; AgRP: Agouti-related peptide; AhR: aryl hydrocarbon receptor; APOA4: apolipoprotein A4; AR: androgen receptor; BAT: brown adipose tissue; BF%: body fat percentage; BMI: body mass index; BPA: bisphenol A; BPB: bisphenol B; BPC: bisphenol C; BPE: bisphenol E; BPAF: bisphenol AF; BPP: bisphenol P; BPZ: bisphenol Z; CB1: endocannabinoid receptor type 1; C/EBP: CCAAT/enhancer-binding family of proteins; CPT1α: carnitine palmitoyltransferase α; CRP: C-reactive protein; DBP: dibutyl phthalate; DCP: 2,4-dichlorophenol; DEHP: di(2-ethylhexyl) phthalate; DEP: diethyl phthalate; DIBP: diisobutyl phthalate; DINP: diisononyl phthalate; DPHP: di(2-propylheptyl) phthalate; E2: 17β-estradiol; EDCs: endocrine-disrupting chemicals; EFSA: European Food Safety Authority; ER: estrogen receptor; ERR-γ: estrogen-related γ receptor; EU: European Union; FABP4: fatty-acid-binding protein 4; FASN: fatty acid synthase; FDA: Food and Drug Administration; GPR30: G-protein-coupled receptor 30; GLUT: glucose transporter; GR: glucocorticoid receptor; GWAS: genome-wide association study; HDL: high-density lipoprotein; HOMA-IR: Homeostatic Model Assessment for Insulin Resistance; HPP: 4-cumylphenol; IFN: interferon; IGF: insulin-like growth factor; IL: interleukin; IR: insulin resistance; JAK: Janus kinase; JNK: c-Jun N-terminal kinase; LBP: lipopolysaccharide-binding protein; LPL: lipoprotein lipase; LPS: lipopolysaccharide; MCP: monocyte chemoattractant protein-1; MEHP: mono-(2-ethylhexyl) phthalate; MR: mineralocorticoid receptor; mTOR: mammalian target of rapamycin; NAFLD: non-alcoholic fatty liver disease; NF-κB: nuclear factor kappa-light-chain-enhancer of activated B cells; NPY: neuropeptide Y; PPAR: peroxisome-proliferator-activated receptors; PR: progesterone receptor; PVC: polyvinyl chloride; ROS: reactive oxygen species; SGBSs: Simpson–Golabi–Behmel syndrome pre-adipocyte cells; SREBF1: sterol regulatory element-binding factor 1; STAT: signal transducer and activator of transcription; T2DM: diabetes mellitus type 2; TBBPA: tetrabromobisphenol A; TBPH: Bis(2-ethyhexyl) tetrabromophthalate; TDI: tolerable daily intake; TG: triacylglycerol; TGF: transforming growth factor; TNF-α: tumor necrosis factor-α; Treg: T regulatory cell; TR: thyroid hormone receptors; TSH: thyroid-stimulating hormone; VEGFA: vascular endothelial growth factor A; WAT: white adipose tissue; WHO: World Health Organization.
